# The Kir2.1^E299V^ mutation increases atrial fibrillation vulnerability while protecting the ventricles against arrhythmias in a mouse model of short QT syndrome type 3

**DOI:** 10.1093/cvr/cvae019

**Published:** 2024-01-23

**Authors:** Ana I Moreno-Manuel, Álvaro Macías, Francisco M Cruz, Lilian K Gutiérrez, Fernando Martínez, Andrés González-Guerra, Isabel Martínez Carrascoso, Francisco José Bermúdez-Jimenez, Patricia Sánchez-Pérez, María Linarejos Vera-Pedrosa, Juan Manuel Ruiz-Robles, Juan A Bernal, José Jalife

**Affiliations:** Centro Nacional de Investigaciones Cardiovasculares (CNIC), Melchor Fernández Almagro 3, 28029 Madrid, Spain; Centro Nacional de Investigaciones Cardiovasculares (CNIC), Melchor Fernández Almagro 3, 28029 Madrid, Spain; Centro Nacional de Investigaciones Cardiovasculares (CNIC), Melchor Fernández Almagro 3, 28029 Madrid, Spain; Centro Nacional de Investigaciones Cardiovasculares (CNIC), Melchor Fernández Almagro 3, 28029 Madrid, Spain; Centro Nacional de Investigaciones Cardiovasculares (CNIC), Melchor Fernández Almagro 3, 28029 Madrid, Spain; CIBER de Enfermedades Cardiovasculares (CIBERCV), Madrid, Spain; Centro Nacional de Investigaciones Cardiovasculares (CNIC), Melchor Fernández Almagro 3, 28029 Madrid, Spain; Centro Nacional de Investigaciones Cardiovasculares (CNIC), Melchor Fernández Almagro 3, 28029 Madrid, Spain; Centro Nacional de Investigaciones Cardiovasculares (CNIC), Melchor Fernández Almagro 3, 28029 Madrid, Spain; Department of Cardiology, Hospital Universitario Virgen de las Nieves, 18014 Granada, Spain; Centro Nacional de Investigaciones Cardiovasculares (CNIC), Melchor Fernández Almagro 3, 28029 Madrid, Spain; Centro Nacional de Investigaciones Cardiovasculares (CNIC), Melchor Fernández Almagro 3, 28029 Madrid, Spain; Centro Nacional de Investigaciones Cardiovasculares (CNIC), Melchor Fernández Almagro 3, 28029 Madrid, Spain; Centro Nacional de Investigaciones Cardiovasculares (CNIC), Melchor Fernández Almagro 3, 28029 Madrid, Spain; CIBER de Enfermedades Cardiovasculares (CIBERCV), Madrid, Spain; Centro Nacional de Investigaciones Cardiovasculares (CNIC), Melchor Fernández Almagro 3, 28029 Madrid, Spain; CIBER de Enfermedades Cardiovasculares (CIBERCV), Madrid, Spain; Departments of Internal Medicine and Molecular and Integrative Physiology, Center for Arrhythmia Research, University of Michigan, Ann Arbor, MI 4810, USA

**Keywords:** Electrocardiogram, Action potential duration, Excitability, Kir2.1-Nav1.5 channelosome, Atrial and ventricular arrhythmias

## Abstract

**Aims:**

Short QT syndrome type 3 (SQTS3) is a rare arrhythmogenic disease caused by gain-of-function mutations in *KCNJ2*, the gene coding the inward rectifier potassium channel Kir2.1. We used a multidisciplinary approach and investigated arrhythmogenic mechanisms in an *in-vivo* model of *de-novo* mutation Kir2.1^E299V^ identified in a patient presenting an extremely abbreviated QT interval and paroxysmal atrial fibrillation.

**Methods and results:**

We used intravenous adeno-associated virus-mediated gene transfer to generate mouse models, and confirmed cardiac-specific expression of Kir2.1^WT^ or Kir2.1^E299V^. On ECG, the Kir2.1^E299V^ mouse recapitulated the QT interval shortening and the atrial-specific arrhythmia of the patient. The PR interval was also significantly shorter in Kir2.1^E299V^ mice. Patch-clamping showed extremely abbreviated action potentials in both atrial and ventricular Kir2.1^E299V^ cardiomyocytes due to a lack of inward-going rectification and increased I_K1_ at voltages positive to −80 mV. Relative to Kir2.1^WT^, atrial Kir2.1^E299V^ cardiomyocytes had a significantly reduced slope conductance at voltages negative to −80 mV. After confirming a higher proportion of heterotetrameric Kir2.x channels containing Kir2.2 subunits in the atria, *in-silico* 3D simulations predicted an atrial-specific impairment of polyamine block and reduced pore diameter in the Kir2.1^E299V^-Kir2.2^WT^ channel. In ventricular cardiomyocytes, the mutation increased excitability by shifting I_Na_ activation and inactivation in the hyperpolarizing direction, which protected the ventricle against arrhythmia. Moreover, Purkinje myocytes from Kir2.1^E299V^ mice manifested substantially higher I_Na_ density than Kir2.1^WT^, explaining the abbreviation in the PR interval.

**Conclusion:**

The first *in-vivo* mouse model of cardiac-specific SQTS3 recapitulates the electrophysiological phenotype of a patient with the Kir2.1^E299V^ mutation. Kir2.1^E299V^ eliminates rectification in both cardiac chambers but protects against ventricular arrhythmias by increasing excitability in both Purkinje-fiber network and ventricles. Consequently, the predominant arrhythmias are supraventricular likely due to the lack of inward rectification and atrial-specific reduced pore diameter of the Kir2.1^E299V^-Kir2.2^WT^ heterotetramer.

## Graphical Abstract

**Graphical Abstract:**
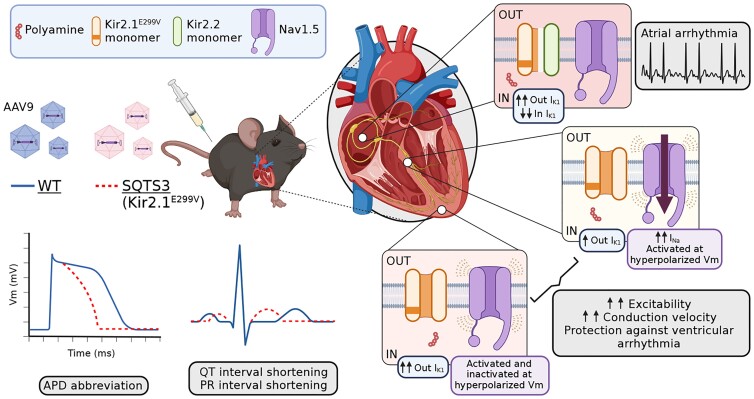

**Time for primary review: 55 days**

**See the editorial comment for this article ‘Atrial fibrillation in the young: consider heritable conditions like short QT syndrome’, by L. Fabritz and M.D. Lemoine, https://doi.org/10.1093/cvr/cvae041.**

## 1. Introduction

Short QT syndrome (SQTS) is a rare, highly lethal inheritable disease characterized by an abnormally short QT interval on the electrocardiogram (ECG) and an increased risk for atrial- and ventricular fibrillation (AF/VF), and sudden cardiac death (SCD).^1–3^ To date, less than 250 cases in nearly 150 families have been diagnosed worldwide, all during the last decades.^1,3^ Despite their heterogeneous phenotype, SQTS patients can manifest palpitations, cardiac arrest, syncope, or AF.^4^

SQTS is considered a disease with an autosomal dominant inheritance. However, only 20–30% of patients with SQTS have an identifiable mutation.^5,6^ To date, only four genes encoding potassium channels (*KCNQ1*, *KCNH2*, and *KCNJ2*) and the chloride-bicarbonate exchanger AE3 (*SLC4A3*) have been clearly associated with pathogenic SQTS.^7–9^ However, we still lack detailed information about the factors responsible for the relative malignancy and specific arrhythmogenic mechanisms of each of the known mutations.

SQTS type 3 [SQTS3 (OMIM 609622)] is caused by *KCNJ2* gain-of-function mutations.^10,11^ The *KCNJ2* gene encodes the strong inward rectifier potassium channel Kir2.1 responsible for I_K1_.^12^ Outward currents through Kir2.x channels regulate the resting membrane potential (RMP), the threshold for excitation, and the final phase of action potential (AP) repolarization.^12,13^ Among the SQTS3 causative mutations, Kir2.1^E299V^ (c.896A > T) was identified in an 11-year-old boy with an extremely abbreviated QT interval (200 ms) and paroxysmal AF, but without ventricular arrhythmias despite mild left ventricular dysfunction (possibly due to the rapid AF rate).^14^ The defects caused by Kir2.1^E299V^ were studied in a heterologous expression system.^14^ While valid, the approach precluded investigating arrhythmogenic mechanisms in the complex cardiac environment. Importantly, glutamic acid at position 299 is highly conserved in Kir2.1 channels among species.^10^ It is located at the cytoplasmic domain and, together with other negatively charged residues, forms the inner vestibule of the channel pore, which determines the strength of inward I_K1_ rectification.^15,16^ Inward rectification is attributed to a voltage-dependent blockade of outward current by internal Mg^2+^ and polyamines (spermine, spermidine, and putrescine).^17^ In Kir2.1 channels, rectification is regulated by two different negatively charged regions, one in the transmembrane domain, involving D172, and the other in the cytoplasmic region, involving E224, D255, D259, and E299.^18^ Polyamines are important in aging, cancer, and other diseases, but induction of inward rectification is likely their most important function.^19^ However, to our knowledge, polyamines have never been used to investigate SQTS3 mechanisms or SCD, and it is unknown whether they have a role in linking channel dysfunction to arrhythmias.

On the other side, Kir2.1 interacts with the cardiac voltage-gated sodium channel Na_V_1.5 forming *channelosomes* from early stages of their common trafficking pathway, and both channels regulate each other’s function.^20^ Trafficking-deficient mutations in one of these channels reduce the surface expression and current density of the other.^21–25^ However, it is unknown whether gain-of-function mutations in one or the other channel modify such interactions or result in unforeseen electrical remodelling mediated by changes in other interacting proteins.

Here, we report on the first *in-vivo* mouse model of cardiac-specific SQTS3 mimicking the electrophysiological phenotype of a patient with the Kir2.1^E299V^ mutation. A clear consequence of the mutation was the extreme atrial and ventricular AP and QT interval shortening due to the loss of polyamine-mediated inward-going rectification. However, unlike the atria, the gain-of-function of this Kir2.1 mutation increased excitability and protected against arrhythmia inducibility in the ventricles. Therefore, like in the patient, the predominant arrhythmias were supraventricular, including atrial tachycardia and AF.

## 2. Methods

Detailed descriptions are provided in the Supplementary material online.

### 2.1 Study approval

All experimental procedures using animals conformed to EU Directive 2010/63EU and Recommendation 2007/526/EC, enforced in Spanish law under *Real Decreto 53/2013*. They were approved by the local ethics committees and the Animal Protection Area of the Comunidad Autónoma de Madrid (PROEX 111.4/20).

### 2.2 Mice

Four-week-old C57BL/6J male mice were obtained from Charles River Laboratories. Mice were reared and housed in accordance with institutional guidelines and regulations.

### 2.3 Adeno-associated virus (AAV) production, injection, and mouse models generation

Vectors encoding wildtype (WT) Kir2.1 (Kir2.1^WT^) or the SQTS3 Kir2.1 mutant (Kir2.1^E299V^) were packaged into adeno-associated virus (AAV) serotype 9 (AVV9) capsids.^26–29^ After anaesthesia (Ketamine 60 mg/kg and Xylazine 20 mg/kg i.p.), 4- to 5-week-old mice were administered 3.5 × 10^10^ viral genomes (vg) per animal i.v. in a final volume of 50μL.^26,30^ Mice were used for experiments at 15–25 weeks of age.

### 2.4 Echocardiography

Mice were anaesthetised with 0.5–2% Isoflurane in oxygen, and placed on a 37°C heating platform in the supine position. Transthoracic echocardiography was performed blindly by an expert operator using a high-frequency ultrasound system (Vevo 2100, VisualSonics Inc., Canada) with a 40-MHz linear probe, and analyzed blindly as described (see Supplementary material online).

### 2.5 Surface ECG recordings

Mice were anaesthetised with 0.8–1% Isoflurane in oxygen. A subcutaneous 23-gauge needle electrode connected to an MP36R amplifier (BIOPAC Systems) was attached to each limb, and six-lead surface ECGs were recorded for 5 min. We analyzed blindly the recordings using AcqKnowledge 4.1 software.

### 2.6 *In-vivo* intracardial electrophysiology

After anaesthesia (Ketamine 60 mg/kg and Xylazine 20 mg/kg i.p.), an octopolar catheter (Science) was inserted in the heart through the jugular vein.^31,32^ Refractory periods and arrhythmia inducibility were assessed in control and mutant mice.

### 2.7 Optical mapping in isolated hearts

Optical mapping experiments in Kir2.1^WT^ and Kir2.1^E299V^ mice were carried out blindly as previously described.^33^

### 2.8 Atrial and ventricular cardiomyocyte isolation

After cervical dislocation, the mouse heart was mounted on a Langendorff-perfusion apparatus, and the aorta was retrogradely perfused, as per Macías *et al*.^34^

### 2.9 HEK-293 T/HEK-Na_V_1.5 cells culture and transfection

We maintained human embryonic kidney (HEK) 293 T (ATCC number CRL-3216) and HEK-Na_V_1.5 cells (kindly provided by Dr. Carmen Valenzuela, CSIC-UAM Madrid) in Dulbecco's modified eagle medium supplemented with 10% fetal bovine serum, 1% penicillin/streptomycin and *L*-glutamine. We used 0.2% Zeocin to select Na_V_1.5 containing cells.^35^ We transfected these cells using JetPRIME transfection reagent (Polyplus).

### 2.10 Patch-clamping of cardiomyocytes and HEK cells

Whole-cell (current- and voltage-clamp), inside-out patch-clamping, and data analysis procedures were as described previously^22–24,36,37^ (see Supplementary material online). External and internal solutions are listed in Supplementary material online, *Table S1*.^24,38^

### 2.11 Immunohistochemistry/fluorescence

We used goat polyclonal anti-Tomato antibody (Sicgen, AB8181-200) and hematoxylin-eosin in 5–7µm-thick sections to analyze blindly mouse heart tissue structure and the level of AAV9 infection.

### 2.12 Immunofluorescence

Isolated cardiomyocytes were processed and incubated with primary and secondary antibodies specified in Supplementary material online, *Tables S2* and *S3*. Images were acquired with a Leica SP8 confocal microscope.

### 2.13 Western blot and membrane fractionation

Whole hearts or specific cardiac chambers from control and mutant mice were excised and lysed using ice-cold radioimmunoprecipitation assay buffer. To measure Kir2.1 membrane protein levels in mouse cardiomyocytes, we followed manufacturer’s instructions (Abcam, ab65400). 25–80 µg of protein resolved in 5–10% SDS-PAGE gels. Antibodies are listed in Supplementary material online, *Tables S4* and *S5*.

### 2.14 Quantitative real-time PCR (qRT-PCR)

Heart samples from uninfected, AAV9-Tomato, AAV9-Kir2.1^WT^, and AAV9-Kir2.1^E299V^ mice were homogenized for RNA extraction. The resultant cDNA was analyzed by qRT-PCR using specific primers to amplify the desired genetic products (see Supplementary material online, *Table S6*).

### 2.15 *In silico* modelling

Protein data bank templates were generated from the Fast-All (FASTA) sequences of Kir2.1, Kir2.2, and Kir2.3.^39^ Comparative modelling was performed using the ROSETTA framework.^40^ The target sequences (FASTA format) including the E299V mutation were threaded onto the three-dimensional backbone of the template structures according to a multi-sequence alignment. See details in the Supplementary material online.

### 2.16 Statistical analysis

We used GraphPad Prism software versions 7.0 and 8.0, normal (Gaussian) distribution analysis (Shapiro–Wilk test), Grubb’s test for outliers, and Student’s *t*-test for comparisons. For non-Gaussian distributions, we applied the nonparametric Mann–Whitney test. We used one- or two-way analysis of variance (ANOVA) for comparison among more than two groups. Data are expressed as mean ± SEM, and differences are considered significant at *P* < 0.05 (*P < 0.05; **P < 0.01; ***P < 0.001; ****P < 0.0001). Note that ‘N’ refers to the number of mice or transfections used and ‘n’ to the number of cells analyzed per mice/transfection.

## 3. Results

### 3.1 Validation and characterization of mouse models

We generated mouse models of SQTS3 using AAV9. We confirmed cardiac-specific expression of Kir2.1^WT^ or Kir2.1^E299V^ by the fluorescence emitted by the tdTomato reporter in the AAV construct, totally absent in uninfected hearts (see Supplementary material online, *Figure S1A and B*). Immunohistochemistry of tdTomato in ventricular and atrial slices confirmed global AAV9 infection of ∼95%, with ∼50% cardiomyocytes expressing 2–4vg/cell (see Supplementary material online, *Figure S1C–F*), as reported previously.^26^ We also confirmed the infection of cardiac conduction system cells using Cx40^GFP^ transgenic mice (see Supplementary material online, *Figure S1G*). Haematoxylin-Eosin staining and echocardiographic analysis confirmed the unaffected structure and contractile function in either Kir2.1^WT^ or Kir2.1^E299V^ hearts (see Supplementary material online, *Figure S1C* and *S2*).

qRT-PCR of the *trans*gene demonstrated that, unlike the uninfected group, hearts from Kir2.1^WT^ and Kir2.1^E299V^ mice amplified the human *KCNJ2* (see Supplementary material online, *Figure S3A*). We also ensured that genetic haploinsufficiency did not operate after expression in *trans* of Kir2.1^WT^ and Kir2.1^E299V^. We confirmed that WT and mutant transcripts did not disturb endogenous Kir2.1–3 mRNA levels (see Supplementary material online, *Figure S3A*). As described,^41^ the Kir2.3 isoform is not predominant in murine myocardium, so the *KCNJ4* mRNA was undetectable in our experiments. Despite the amplification of the human *KCNJ2* confirmed by qRT-PCR, total protein levels from the whole hearts of uninfected, Kir2.1^WT^ and Kir2.1^E299V^ were very similar (see Supplementary material online, *Figure S3B*, left panel, and Supplementary material online, *Figure S4*). Endogenous regulatory mechanisms keep Kir2.1 translation at the same levels as when there is no infection, without overexpression in the generated models. Surface membrane Kir2.1 levels in Kir2.1^WT^ and Kir2.1^E299V^ hearts were also similar to each other, so the gain-of-function caused by the E299V mutation was not due to a higher protein expression (see Supplementary material online, *Figure S3B*, right panel, and Supplementary material online, *Figure S4*). Kir2.1 channels placed at the membrane present three bands on western blot likely due to their different glycosylation levels depending on the trafficking pathway.^23^ Therefore, since AAV9-Kir2.1^WT^ did not alter the Kir2.1 total expression and represented an infected control, all subsequent experiments were conducted in Kir2.1^E299V^ animals and compared with Kir2.1^WT^ controls.

### 3.2 Kir2.1^E299V^ mice have an extremely short QT interval

**Figure 1:**
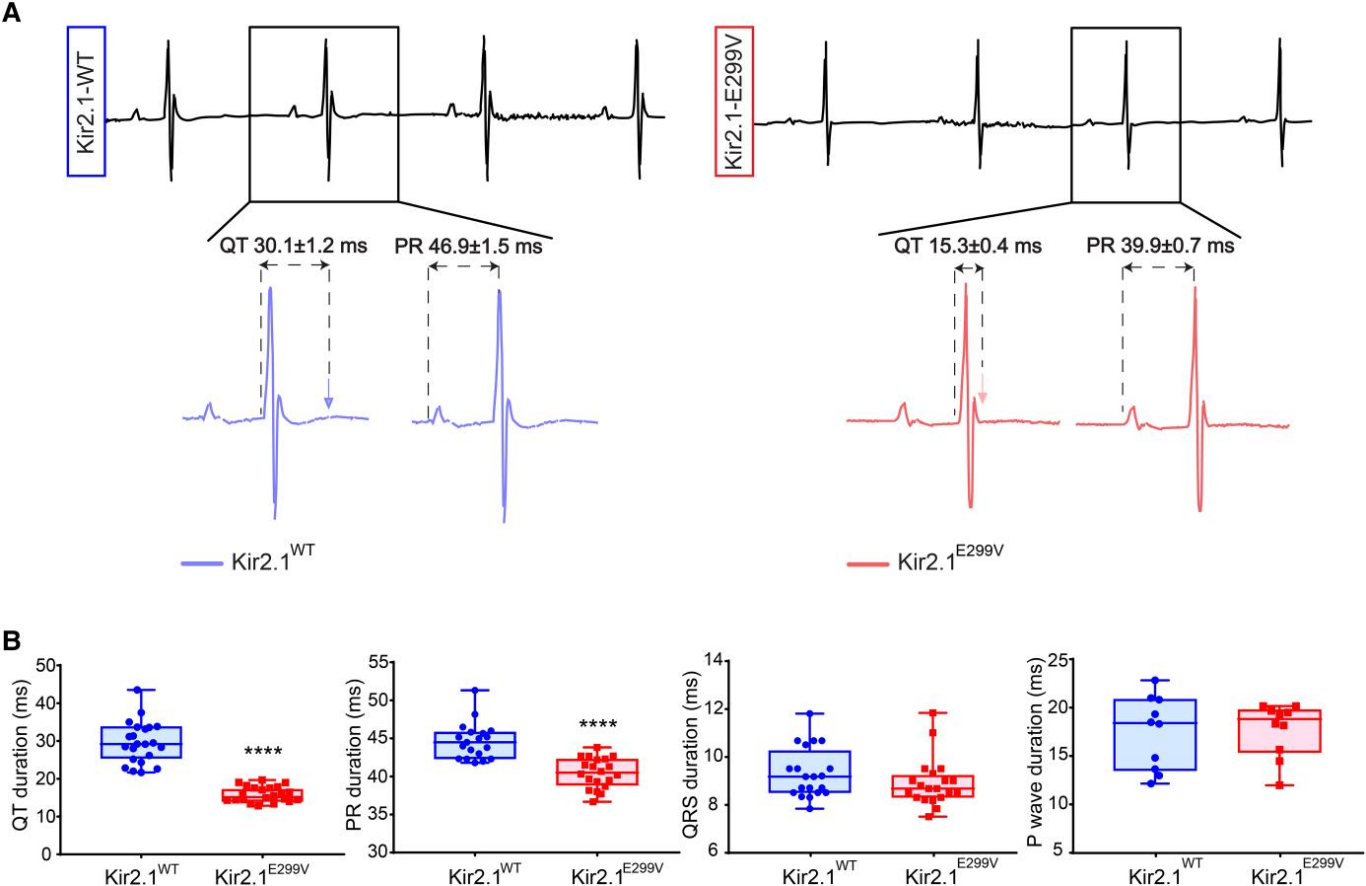
The Kir2.1^E299V^ mouse model reproduces the ECG phenotype of SQTS3. *A*, Representative ECG traces showing extreme abbreviation of QT and PR intervals in Kir2.1^WT^ (left) vs. Kir2.1^E299V^ (right) mice. *B*, Quantification of the QT interval (15.3 ± 0.4 ms vs. 30.1 ± 1.2 ms) (****P < 0.0001; *N* = 30 (WT), 28 (E299V)), PR interval (39.9 ± 0.7 ms vs. 46.9 ± 1.5 ms) (****P < 0.0001; *N* = 20), the QRS complex (8.9 ± 0.2 ms vs. 9.3 ± 0.2 ms) (*P* = 0.2961; *N* = 20), and *P*-wave duration (17.7 ± 0.9 ms vs. 17.4 ± 1.2 ms) (*P* = 0.9995; *N* = 10) in Kir2.1^E299V^ and Kir2.1^WT^, respectively. Unpaired two-tailed Students’ *t*-test (QT and *P*-wave) or Mann–Whitney test (PR interval and QRS complex) were used.

Under basal conditions, both the non-corrected and the corrected QT (QTc) intervals were significantly shorter in Kir2.1^E299V^ than in Kir2.1^WT^ animals (*Figure 1A*, and Supplementary material online, *Figures S5* and *S6*). The PR interval was also significantly shorter in mutant than WT animals. We used isoproterenol (ISO, 5 mg/kg) to study the response of our models to a stress situation in which the heart rate increases. ISO prolonged the QT interval in Kir2.1^E299V^ to levels similar to Kir2.1^WT^, but the PR interval continued to reduce in mutant mice (see Supplementary material online, *Figure S7A*). Both P-wave duration and QRS complex were similar in both groups at baseline, although the QRS tended to be shorter in mutant animals (*Figure 1B*). Notably, i.p. administration of ISO further shortened the QRS in Kir2.1^E299V^ mice making it significantly different from WT (see Supplementary material online, *Figure S7A*). Together these data indicated that, in addition to the QT abbreviation characteristic of SQTS3, Kir2.1^E299V^ animals had increased atrio-ventricular (AV) and intraventricular conduction velocities (CV), particularly in the presence of ISO.

Chloroquine and flecainide inhibit I_Kr_ and may be beneficial for atrial arrhythmia in patients carrying the Kir2.1^E299V^ mutation.^42^ Chloroquine selectively blocks Kir2.1 channels at <10 µM.^43–46^ In Kir2.1^E299V^ mice, chloroquine (40 mg/Kg corresponding to <10 µM in blood when administered i.p.^47–49^) induced an initial rapid prolongation (<1 min) that brought the QT interval to the Kir2.1^WT^ mouse level. Unfortunately, there was a constant and progressive QT prolongation over a 40-min period in both groups. Chloroquine also prolonged the PR and QRS intervals to non-physiologic durations (see Supplementary material online, *Figure S7B*), highlighting the potential risk of using chloroquine to treat SQTS3 patients. To study if flecainide could normalize the ECG values in Kir2.1^E299V^ animals, we tested the response of both mouse models to this class 1c antiarrhythmic drug (20 mg/kg). Flecainide blocks the cardiac sodium channel and has other effects on excitation–contraction coupling and potassium channels.^50–52^ As demonstrated in Supplementary material online, *Figure S7C*, flecainide produced a relatively rapid but transient prolongation in the QT interval of the Kir2.1^WT^ mice, but had no effect whatsoever on the QT interval of Kir2.1^E299V^ mice. In contrast, the drug gradually prolonged the PR and QRS intervals over a 6-min period in both groups. In summary, unlike flecainide, chloroquine effectively prolongs the QT interval. However, the effects of both drugs on AV and intraventricular conduction make them unlikely candidates for antiarrhythmic therapy in SQTS3 patients. The beneficial effects of these drugs would not be reflected in mice because their hearts do not express I_Kr_.

### 3.3 Kir2.1^E299V^ mouse hearts have shorter refractory periods than WT and are highly inducible for atrial but not ventricular arrhythmias

**Figure 2:**
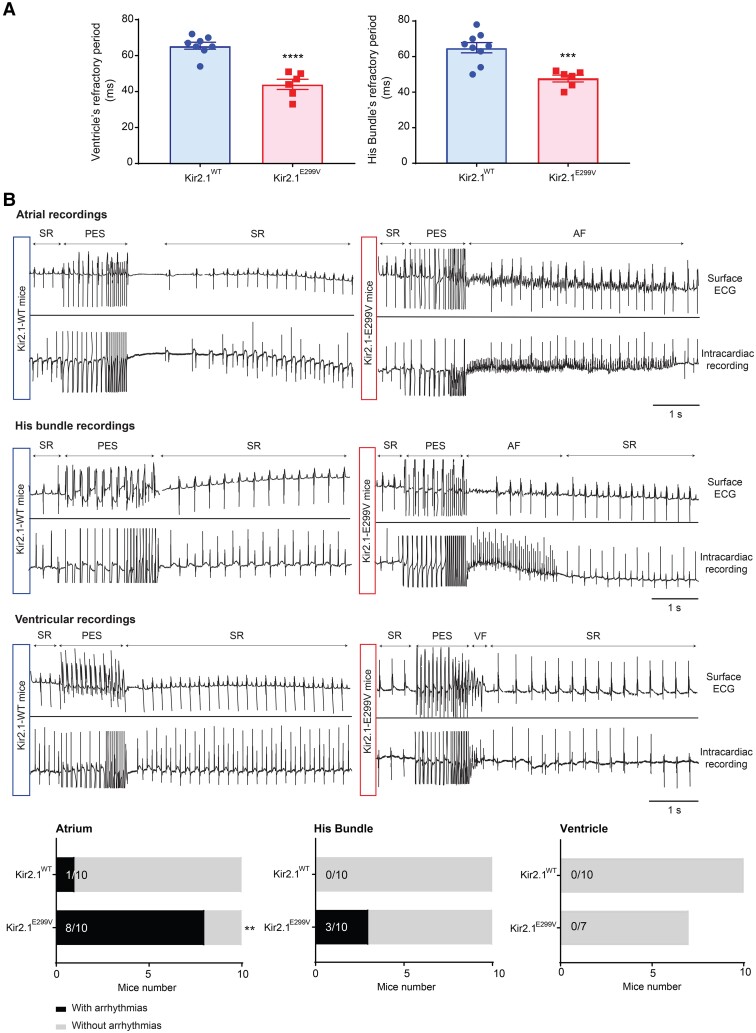
Kir2.1^E299V^ mice have a short refractory period and are highly inducible for atrial arrhythmias. *A*, Ventricular refractory period of Kir2.1^WT^ (*N* = 8) and Kir2.1^E299V^ (*N* = 6) (65.5 ± 1.9 ms and 44 ± 2.8 ms, respectively; ****P < 0.0001). His bundle stimulation yielded a refractory period of 65 ± 2.9 ms in Kir2.1^WT^ vs. 47.7 ± 1.9 ms in Kir2.1^E299V^ (***P = 0.0006; *N* = 9 (WT), 6 (E299V)). *B*, Top, Simultaneous surface ECG and intracardiac recordings from right atrium, His bundle, and ventricle before, during, and after PES protocols. Sinus rhythm (SR), PES, and atrial and ventricular fibrillation (AF, VF) are indicated as corresponded in the traces. Bottom, quantification of atrial tachycardia or AF events lasting >1 s after atrial stimulation in left (1 out of 10 in Kir2.1^WT^ mice, and 8 out of 10 in Kir2.1^E299V^) (**P = 0.0055; *N* = 10), His bundle stimulation in the middle (only 3 out of 10 Kir2.1^E299V^), and ventricular stimulation in right. Unpaired 2-tailed Student’s *t*-test (refractory periods) and Fisher’s exact test (presence/absence arrhythmias) were applied.

We measured refractory periods in both mouse groups by stimulating the His bundle (localized as described in Alanís *et al*.^53^; see Supplementary material online, *Figure S8*) or the right ventricle (RV) on intracardiac programmed stimulation experiments. In Kir2.1^E299V^ mice, the His bundle and the RV refractory periods were 26.6% and 32.8% shorter than control, respectively (*Figure 2A*), which was consistent with the reduced QT interval in the SQTS3 animals.

To test the susceptibility of the SQTS3 model to AF and ventricular tachycardia/fibrillation (VT/VF), we performed intracardiac pacing experiments in both groups of mice by applying an S1-S2 train of 10 and 20 Hz (see Supplementary material online, *Extended Materials and Methods*). Arrhythmia episodes lasting >1 s in atria were higher in mutant animals compared to control. While only 1/10 Kir2.1^WT^ animals were inducible, 8/10 Kir2.1^E299V^ mice manifested atrial tachycardia or >1 s AF episodes (*Figure 2B*). As shown in Supplementary material online, *Figure S9*, 9/10 Kir2.1^E299V^ animals manifested >500 ms atrial arrhythmias episodes.

No Kir2.1^WT^ and only 3/10 Kir2.1^E299V^ animals were inducible for > 1 s arrhythmias (*Figure 2B*) and >500 ms episodes (see Supplementary material online, *Figure S9*) by His bundle stimulation.

On ventricular stimulation, none of the animals were inducible for arrhythmias lasting >1 s, regardless of genotype (*Figure 2B*). However, 3/7 Kir2.1^E299V^ mice had >500 ms ventricular episodes (see Supplementary material online, *Figure S9*).

Even when we stimulated the RV or His bundle of Kir2.1^E299V^ mice, the main type of arrhythmia triggered was AF, as 40% mutant animals yielded atrial arrhythmias when stimulating the RV. Similarly, 60% of Kir2.1^E299V^ animals manifested AF upon His bundle stimulation. Altogether, the vast majority of inducible arrhythmias in the Kir2.1^E299V^ mice were atrial, and of these, the most common was AF. To rule out possible structural alterations underlying the atrial-specific arrhythmia inducibility, we analyzed histologically the atria of WT and mutant animals. We saw no differences in size, wall thickness, or fibrosis (see Supplementary material online, *Figure S10*).

### 3.4 Action potential duration (APD) is extremely brief in both atrial and ventricular cardiomyocytes of Kir2.1^E299V^ mice

**Figure 3:**
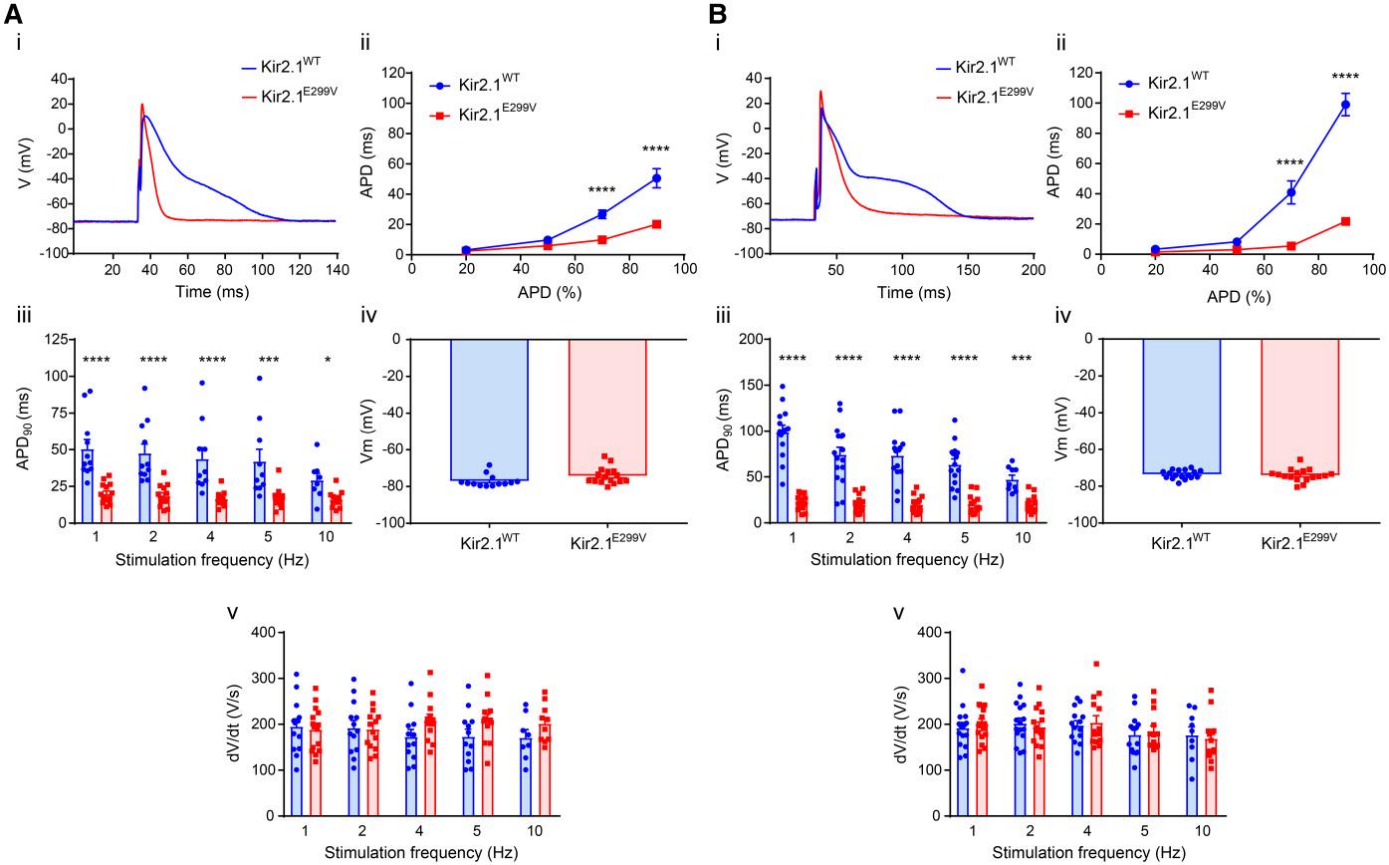
APD is reduced in the atria and ventricles of Kir2.1^E299V^ mice. Electrophysiological characterization of AP from Kir2.1^WT^ and Kir2.1^E299V^. *A*, Atrial cardiomyocytes. Kir2.1^WT^ (*N* = 3, *n* = 8–13) and Kir2.1^E299V^ (*N* = 3, *n* = 10–16). *B*, Ventricular cardiomyocytes. Kir2.1^WT^ (*N* = 3; *n* = 9–17) and Kir2.1^E299V^ (*N* = 3; *n* = 12–17). Different panels in both regions show representative APs recorded at 1 Hz (i), and APDs at 20, 50, 70 (****P < 0.0001) and 90% (****P < 0.0001) of repolarization for 1 Hz pacing (ii). APD_90_ at 1, 2, 4, 5, and 10 Hz is shown in panels iii (****P < 0.0001, ***P < 0.001, *P < 0.05). The RMP for each type of cardiomyocyte (panels iv) and the maxim upstroke velocity (dV/dt_max_) (panels v) are shown. Unpaired two-tailed Students’*t*-test or Mann–Whitney test were applied.

Despite their extremely short QT interval and ventricular refractory period, Kir2.1^E299V^ mice are much less susceptible to ventricular than atrial arrhythmias. This fact leads to the question of whether the mutation results in a more severe electrical phenotype in atrial than ventricular cardiomyocytes. We, therefore, conducted whole-cell current-clamp experiments to measure the AP characteristics. Clearly, the mutation consistently and significantly abbreviated the atrial action potential duration (APD) at all levels and frequencies studied (*Figure 3A*, Supplementary material online, Figure 1*1A* and *Table S7*). As shown in *Figure 3Bi-iii*, the ventricular APDs in Kir2.1^E299V^ cardiomyocytes were also abbreviated to similar levels as the mutant atrial APDs (see Supplementary material online, *Table S8*). The significant shortening of both Kir2.1^E299V^ atrial and ventricular APDs was independent of the stimulation frequency and did not vary too much as they increased. As seen in *Figure 3Aiv-V* and *Biv-V*, and Supplementary material online, *Figure S11*, neither RMP, dV/dt, or AP amplitude (APA) were affected in either chamber. Therefore, while APD was the only parameter modified, it did not explain the significantly different atrial vs. ventricular arrhythmia inducibility caused by the mutation.

### 3.5 I_K1_ is differently affected in atrial vs. ventricular Kir2.1^E299V^ cardiomyocytes

**Figure 4:**
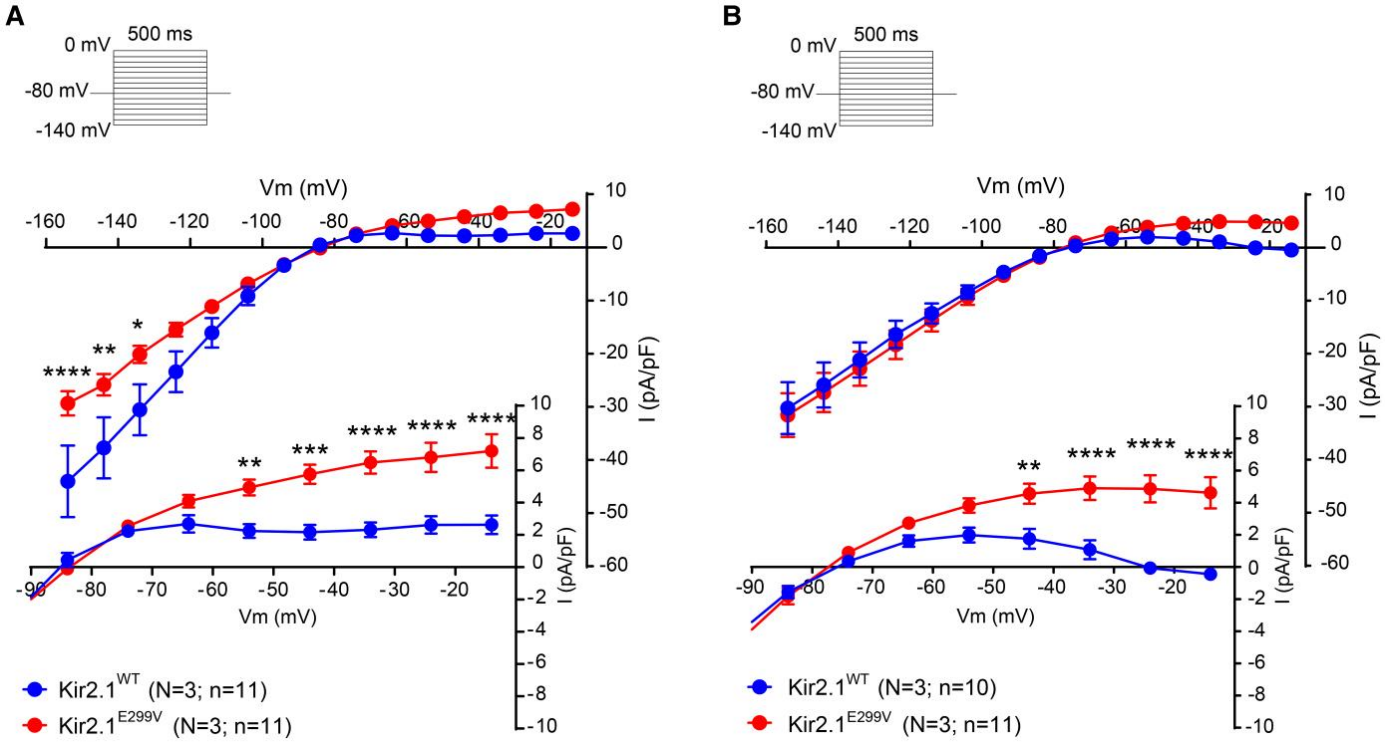
Kir2.1^E299V^ increases outward I_K1_ in both atria and ventricle, but reduces the slope conductance only in the atria. *A*, Atrial cardiomyocytes. Current/voltage (IV) relationships for Kir2.1^WT^ (*N* = 3, *n* = 11) and Kir2.1^E299V^ (*N* = 3; *n* = 11). Note the lack of inward-going rectification with increased outward I_K1_ at voltages positive to −60 mV (7.19 ± 1.04 pA/pF in E299V vs. 2.62 ± 0.57 pA/pF in WT, results at −14 mV; ****P < 0.0001) and loss of inward current at voltages negative to −120 mV (***P < 0.001, **P < 0.01, *P < 0.05 from −135 mV to −160 mV). *B*, Ventricular cardiomyocytes. I_K1_ IV relationships for both experimental groups Kir2.1^WT^ (*N* = 3, *n* = 10) and Kir2.1^E299V^ (*N* = 3; *n* = 11). Lack of inward-going rectification can be appreciated at voltages positive to −50 mV (4.62 ± 0.89 pA/pF in E299V vs. −0.41 ± 0.14 pA/pF in WT, results at −14 mV; ****P < 0.0001) without changes in inward current. Two-way ANOVA was applied for comparisons.

We conducted whole-cell voltage-clamp experiments in atrial and ventricular Kir2.1^WT^ and Kir2.1^E299V^ cardiomyocytes to compare their respective barium-sensitive inward rectifier (I_K1_) current/voltage (IV) relationships. In *Figure 4*, Kir2.1^E299V^ cardiomyocytes from both chambers showed a clear increase in I_K1_ outward current due to loss of inward-going rectification. The outward I_K1_ increase was the mechanism that caused significant APD abbreviation at all the stimulation frequencies in cardiomyocytes from both chambers of the Kir2.1^E299V^ mice (*Figure 3A* and *B*). However, there was an important difference in the effect of the mutation on the I_K1_ IV relation of atrial vs. ventricular cardiomyocytes. While in atrial Kir2.1^E299V^ cardiomyocytes inward I_K1_ was significantly reduced at voltages negative to −80 mV (*Figure 4A*), in the ventricles the inward currents were similar in these ranges of voltage in both genotypes (*Figure 4B*). In other words, inward rectification is absent in both atrial and ventricular Kir2.1^E299V^ cardiomyocytes, but the reduction in the slope conductance of the inward I_K1_ is atrial-specific. This significantly reduced slope conductance relative to WT is likely to predispose the mouse atria to an arrhythmogenic phenotype.

We used *in-silico* modelling to investigate the mechanism of the absence of inward-going rectification in atrial and ventricular Kir2.1^E299V^ cardiomyocytes. First, we determined structural changes in the ventricular isoforms of Kir2.1^WT^ homotetramers (4 Kir2.1^WT^ subunits) and Kir2.1^WT^-Kir2.1^E299V^ heterotetramers (2 Kir2.1^WT^ plus 2 Kir2.1^E299V^ subunits). We observed that mutant heterotetrameric conformations undergo conformational changes, particularly on the lateral chains (Root mean square deviation value of 4.081Å when comparing Kir2.1^WT^-Kir2.1^E299V^ vs. Kir2.1^WT^). From the stability point of view, all the models were stable with very negative values of energy: Kir2.1^WT^ −4801.404 Rosseta Energy Units (REU) and Kir2.1^WT^-Kir2.1^E299V^ −5234.111 REU.

### 3.6 K^+^ ions pass more efficiently through the Kir2.1^WT^-Kir2.1^E299V^ channel structure

**Figure 5:**
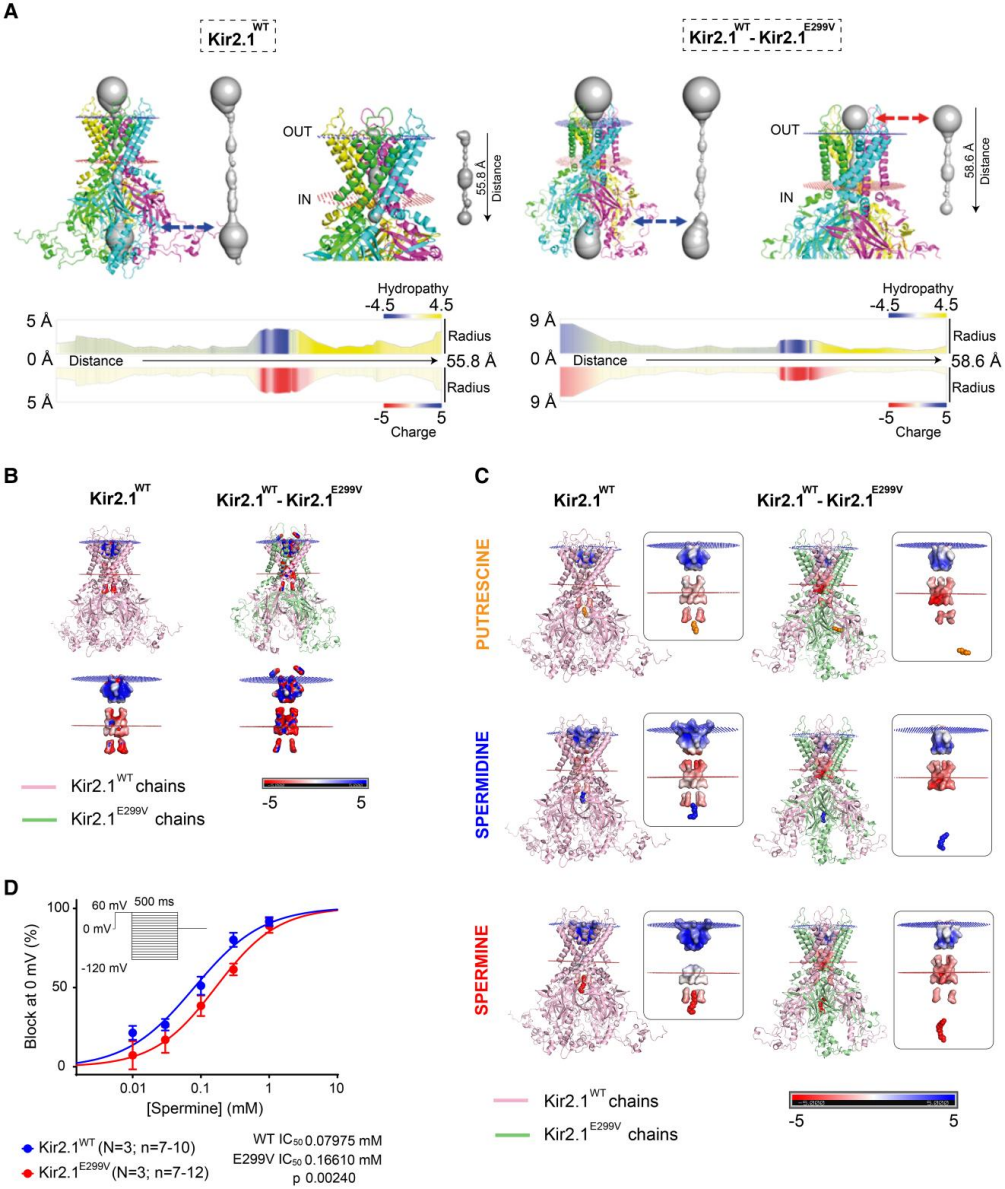
Polyamines fail to block Kir2.1^E299V^ channels. *A*, Top, *In-silico* models of tetrameric structure and pore conformation of Kir2.1^WT^ and Kir2.1^WT^-Kir2.1^E299V^. Bottom, Hydropathy, and charge maps along the extension of the pore. The cytoplasmic Kir2.1^WT^-Kir2.1^E299V^ pore diameter is represented at the bottom of the panel and is appreciably modified by rearrangement of the side chains of residues lining the pore (dashed arrows). In contrast, the extracellular Kir2.1^WT^-Kir2.1^E299V^ pore region undergoes significant expansion and becomes more hydrophilic (right, dashed arrows and shadows). Note the different scales in both bottom panels. *B*, Positive and negative charges distribution analysis of Kir2.1^WT^ vs. Kir2.1^WT^-Kir2.1^E299V^. *C*, Interaction of Kir2.1 with polyamines (putrescine, spermidine, and spermine). *D*, Spermine concentration-response curves in inside-out patch-clamp experiments (IC_50_ 0.07975 mM and 0.1661 mM for Kir2.1^WT^ and Kir2.1^E299V^, respectively) (**P = 0.0024; *N* = 3, *n* = 7–12). *P* value obtained after non-linear fit analysis for comparing IC_50_.

Compared with Kir2.1^WT^, the cytoplasmic pore diameter of the predominant mutant isoform in the ventricles, Kir2.1^WT^-Kir2.1^E299V^, was substantially modified by rearrangement of the side chains of the residues lining the pore (*Figure 5A*, blue dashed arrows). On the other hand, while the transmembrane pore region did not show any appreciable differences between models, the extracellular pore region of the heterotetrameric Kir2.1^WT^-Kir2.1^E299V^ channel underwent significant expansion and became more hydrophilic compared to the homomeric Kir2.1^WT^ (*Figure 5A*, right panel, red discontinue arrows), suggesting that K^+^ ions could pass more efficiently through the mutant channel. Moreover, we saw relevant divergences in charge distribution for each channel, Kir2.1^E299V^ subunits being the most polarized and conducting, contributing to the gain-of-function (*Figure 5B*).

### 3.7 Polyamines fail to block Kir2.1 channels containing the E299V isoform

E299 is one of the cytoplasmic residues that interact with polyamines to confer strong inward rectification.^54,55^ To determine how the E299V mutant modifies I_K1_ rectification, we conducted molecular docking experiments that included the three principal polyamines (putrescine, spermine, and spermidine) along with Kir2.1^WT^ and Kir2.1^WT^-Kir2.1^E299V^. All three polyamines penetrate and block Kir2.1^WT^ channels, but they fail to penetrate the cytoplasmic pore of the Kir2.1^WT^-Kir2.1^E299V^ heterotetramer (*Figure 5C* and Supplementary material online, *Figure S12*). Therefore, the channel remains open at voltages at which it should be closed (voltages positive to −80 mV, *Figure 4*). Notably, the respective electro-potential data show how the docking of polyamines changes the charge distribution significantly from one Kir2.1 conformation to the other. These data also show how the polyamine is far from reaching its binding site at the heterotetrameric Kir2.1^WT^-Kir2.1^E299V^, leaving the channel more polarized and presumably allowing ions to pass through (*Figure 5C*). Altogether, these models help us understand the lack of rectification of mutant Kir2.1^E299V^ channels.

To translate these *in-silico* results to procedures in a realistic environment, we conducted inside-out patch-clamp experiments in Kir2.1^WT^ and Kir2.1^E299V^ ventricular cardiomyocytes, exposing the cytoplasmic side of the channels to different concentrations of spermine. We saw that the spermine concentration needed to block 50% of channels containing Kir2.1^E299V^ subunits was higher than for Kir2.1^WT^ homotetramers (*Figure 5D*). Therefore, the sensitivity of the mutant channels to the polyamine was significantly lower than WT.

### 3.8 A different proportion of Kir2.x subunits in atria vs. ventricles explains the atrial-specific reduction in I_K1_ inward current and the arrhythmia inducibility

**Figure 6:**
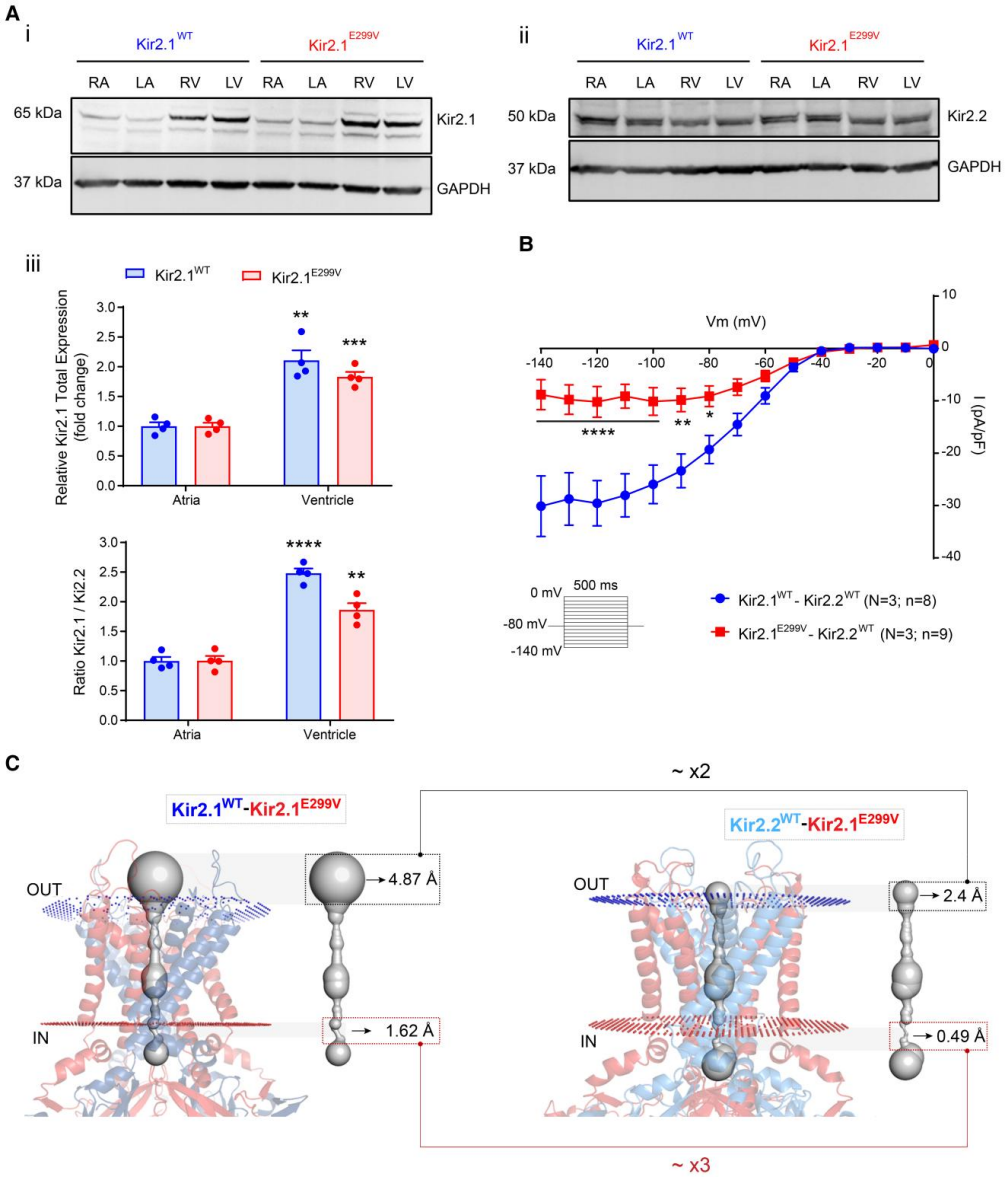
Abundance of Kir2.2 reduces the slope conductance of the inward I_K1_ in atrial Kir2.1^E299V^ channels. A, Western blots for Kir2.1 and Kir2.2 protein levels in atrial and ventricular tissue samples from mice comparing the Kir2.1 (*i*) and Kir2.2 (*ii*) expression levels. (*iii*) Quantification of total Kir2.1 protein levels (top) and Kir.1/Kir2.2 ratio (bottom) in atria vs. ventricles from Kir2.1^WT^ and Kir2.1^E299V^ animals (**P < 0.01, ***P < 0.001 and ****P < 0.0001; duplicate experiments in *N* = 4 animals per condition; Glyceraldehyde-3-phosphate dehydrogenase (GAPDH) was used as loading control for all comparisons). *B*, IV relationship of I_K1_ in HEK-293 cells transfected with dimers expressing Kir2.1^WT^-Kir2.2^WT^ or Kir2.1^E299V^-Kir2.2^WT^ (*N* = 3 independent transfections; *n* = 8–9 cells per condition; *P* < 0.05 for voltages negative to −80 mV). We used a modified external solution (30 mM KCl and 110 mM NaCl) to promote the I_K1_ current, shifting the reversal potential towards more positive voltages (from −80 to −30 mV). C, *In-silico* simulations of Kir2.1^WT^-Kir2.1^E299V^ compared to Kir2.1^E299V^-Kir2.2^WT^ (2.4Å vs. 4.87Å in the extracellular pore of Kir2.1^E299V^-Kir2.2^WT^ and Kir2.1^WT^-Kir2.2^WT^, respectively; 0.49Å vs. 1.62Å in the cytoplasmic pore for Kir2.1^E299V^-Kir2.2^WT^ and Kir2.1^WT^-Kir2.1^E299V^, respectively). We applied Welch’s *t*-test and two-way ANOVA for comparisons.

As shown above, the atria are clearly more susceptible to arrhythmias than the ventricles of the Kir2.1^E299V^ mouse, which correlates with different chamber-specific I_K1_ IV relations. Since both Kir2.1 and Kir2.2 isoforms contribute to I_K1_ in the heart^56^ and it is known that these channels express differently in the atria vs. ventricles,^22,56–61^ we assessed whether different Kir2.x isoform proportions in the atria vs. the ventricle help explain the arrhythmogenic differences we observed. We first measured total Kir2.1 and Kir2.2 protein levels in atria vs. ventricles from mouse samples by western blot. We confirmed that Kir2.1 is highly expressed in the ventricles of both WT and mutant animals, whereas Kir2.1/Kir2.2 levels are close to 1 in mouse atria (*Figure 6A* and Supplementary material online, *Figure S13*). Therefore, a higher proportion of Kir2.1-Kir2.2 heterotetramers should be conducting I_K1_ in atria compared with ventricles, which possibly explained the chamber-specific differences in the pro-arrhythmic effects of the Kir2.1^E299V^ mutant channels.

To evaluate the above hypothesis, we transfected HEK-293 T cells with non-viral piggy-bac vectors encoding the designed dimers Kir2.1^WT^-Kir2.2^WT^ and Kir2.1^E299V^-Kir2.2^WT^ as fusion proteins. As shown in *Figure 6B*, cells transfected with Kir2.1^E299V^-Kir2.2^WT^ reproduced the reduced I_K1_ inward component of atrial Kir2.1^E299V^ cardiomyocytes (see *Figure 4A*). Cells expressing Kir2.1^E299V^-Kir2.2^WT^ channels had a reduced slope conductance at voltages negative to −60 mV for these specific experimental conditions (30 mM KCl and 110 mM NaCl in the external solution to promote I_K1_ density, shifting the reversal potential from −80 to −30 mV). To assess how these changes in the atria should occur structurally, we conducted additional *in-silico* modelling of Kir2.2 heterotetramers. Mutant channels containing Kir2.1^E299V^-Kir2.2^WT^ subunits had a reduced pore diameter and modifications in their polarity (see Supplementary material online, *Figure 1^4^A*). In addition, unlike the Kir2.2^WT^ homotetramers, the Kir2.1^E299V^-Kir2.2^WT^ heterotetramers were unable to bind polyamines or rectify properly (see Supplementary material online, *Figure 1^4^B*). Moreover, analysing specifically the values, in the absence of polyamines, the pore diameter of Kir2.1^E299V^-Kir2.2^WT^ was smaller than Kir2.1^E299V^-Kir2.1^WT^ channels (2.4Å vs. 4.87Å in the extracellular pore, and 0.49Å vs. 1.62Å in the cytoplasmic pore, respectively) (*Figure 6C*). Altogether, these data suggest that the reduced pore diameter of mutant Kir2.1^E299V^-Kir2.2^WT^ channels in atria led to relatively lower atrial than ventricular I_K1_ conductance and may underlie, at least in part, the greater arrhythmogenic potential of the atria of E299V animals.

### 3.9 The Kir2.1^E299V^ mutation increases ventricular excitability by modifying Na_V_1.5 function

**Figure 7:**
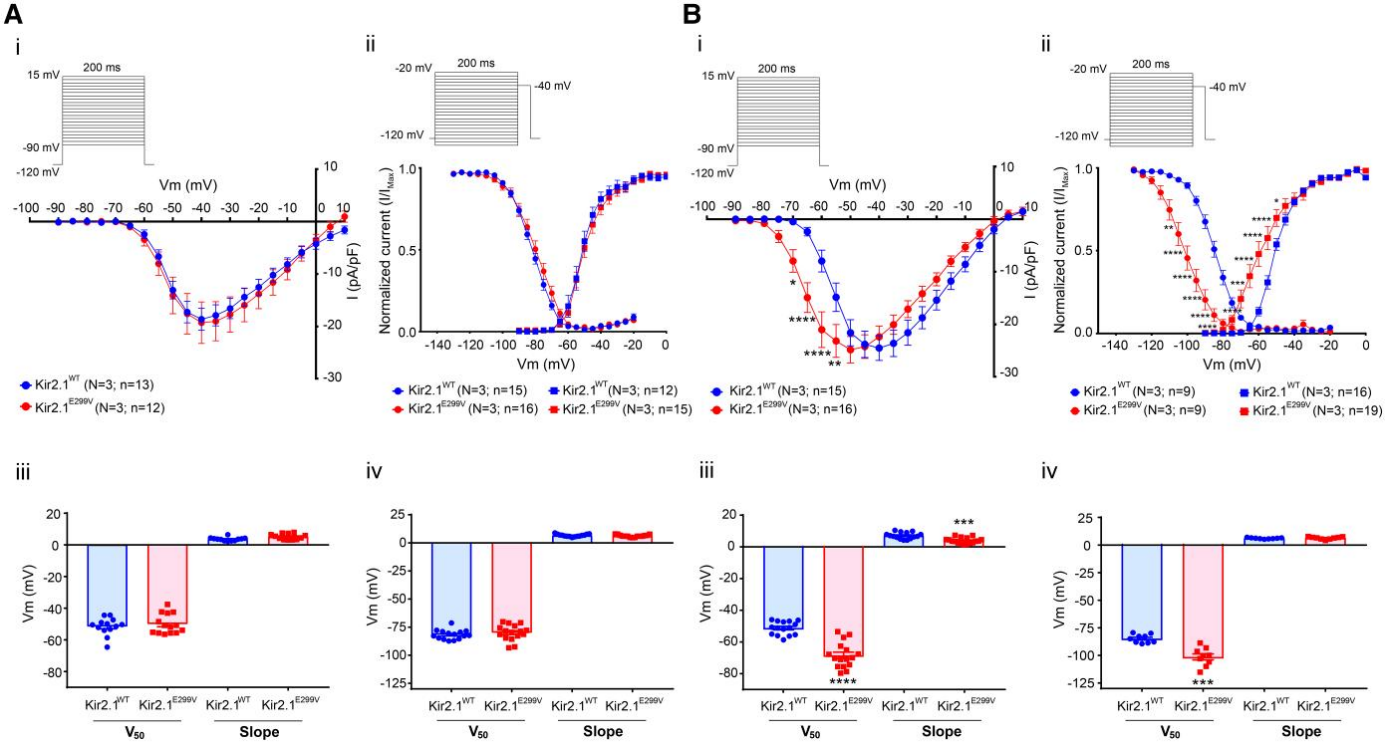
Kir2.1^E299V^ modifies sodium current properties in ventricular but not atrial cardiomyocytes. Electrophysiological characterization of sodium currents (I_Na_) from Kir2.1^WT^ and Kir2.1^E299V^ isolated cardiomyocytes. *A*, Atrial cardiomyocytes. Kir2.1^WT^ (*N* = 3, *n* = 13–15) and Kir2.1^E299V^ (*N* = 3, *n* = 12–16). *B*, Ventricular cardiomyocytes. Kir2.1^WT^ (*N* = 3, *n* = 9–16) and Kir2.1^E299V^ (*N* = 3, *n* = 9–19). (*i*) Different panels in both regions show the I_Na_ IV relationships (***P < 0.001, **P < 0.01, and *P < 0.05 for voltages from −70 to −55 mV when comparing WT and mutant ventricular cardiomyocytes). (*ii*) I_Na_ inactivation and activation curves. Note the shift to negative voltages in the I_Na_ inactivation and activation curves of Kir2.1^E299V^ ventricular cardiomyocytes (****P < 0.0001, ***P < 0.001, **P < 0.01, and *P < 0.05). The activation (****P < 0.0001 and ***P < 0.001 when comparing WT and mutant ventricular cardiomyocytes) (*iii*) and inactivation (***P > 0.001 also in WT vs. mutant ventricular myocytes) (*iv*) parameters (V_50_ and slope) are also indicated. Two-way ANOVA and Mann–Whitney test were applied for comparisons.

The ECG of the SQTS3 mouse model showed substantial abbreviation in the PR interval and a slight shortening of the QRS complex, suggesting the possibility of accelerated AV and intraventricular conduction, respectively. This might be due to sodium inward current (I_Na_) modification induced by the Kir2.1^E299V^ mutation.^21,34^ In an additional group of experiments, we measured I_Na_ in atrial and ventricular cardiomyocytes. In *Figure 7A* superimposed IV relations (Ai), and activation and inactivation curves (Aii) from Kir2.1^WT^ and Kir2.1^E299V^ atrial cardiomyocytes showed that the mutation did not modify either I_Na_ density or its biophysical properties (Aiii and Aiv). In *Figure 7B*, the results were completely different for ventricular cardiomyocytes, where the mutation led to a large and significant shift of I_Na_ peak density to the left (Bi) and an equal shift of both activation and inactivation to negative voltages (*Figure 7Bii-iv*).

To study if the above effects resulted in accelerated conduction in mutant ventricles, we performed optical mapping experiments (see Supplementary material online, *Figure S15*). Mean ventricular CV was higher in Kir2.1^E299V^ than Kir2.1^WT^ hearts. Ventricular CV in Kir2.1^E299V^ was also higher than both left and right atria from Kir2.1^WT^ and Kir2.1^E299V^ hearts, which were similar to WT ventricles. These data provide proof that by increasing excitability in ventricular cardiomyocytes, the Kir2.1^E299V^ mutation also increases CV, which likely protects the ventricles against the initiation and maintenance of arrhythmias.

Further analysis of Kir2.1-Na_V_1.5 *channelosome*-related proteins^20,34^ confirmed similar expression levels and distribution patterns of Kir2.1, Na_V_1.5, α1-Syntrophin, and SAP97 between groups (see Supplementary material online, *Figures S16* and *S17*), suggesting that chamber-specific changes in the biophysical properties of I_Na_ were independent of *channelosome* trafficking or scaffolding, at least at the confocal resolution limit.

To investigate more about the mechanisms underlying the differential I_Na_ changes in ventricular vs. atrial Kir2.1^E299V^ cardiomyocytes, we used HEK-293 T cells stably expressing Na_V_1.5 (HEK-Na_V_1.5 cells).^35^ We transfected Kir2.1^WT^ and Kir2.1^E299V^ to simulate a mutant heterozygous condition in HEK-Na_V_1.5 cells. We saw no differences in I_Na_ density between WT vs. mutant conditions as we had in atrial cardiomyocytes (see Supplementary material online, *Figure S18*). In addition to the interactors mentioned above, β subunits play important roles in the regulation of Na_V_1.5 trafficking and function.^35,62–66^ Na_V_β2 and Na_V_β4 are Na_V_1.5 regulatory subunits differently expressed in atria vs. ventricles, having a higher presence in murine ventricles.^64,67,68^ Hence, in HEK-Na_V_1.5 cells expressing Kir2.1^WT^ and Kir2.1^WT/E299V^, we transfected β subunits individually and in combination. The most promising results were obtained in cells expressing Kir2.1^WT/E299V^ + Na_V_β4. While we did not observe any changes in inactivation, we saw a slight shift of activation to negative voltages and an increase in I_Na_ density (see Supplementary material online, *Figure S18*). Surely other interactors are likely involved, but Na_V_β4 appears to be contributing in some way to the Kir2.1^E299V^-mediated modification of Na_V_1.5 properties in the ventricles where this subunit is highly expressed.^68^

### 3.10 The Kir2.1^E299V^ mutation increases I_Na_ density in Purkinje cardiomyocytes

**Figure 8:**
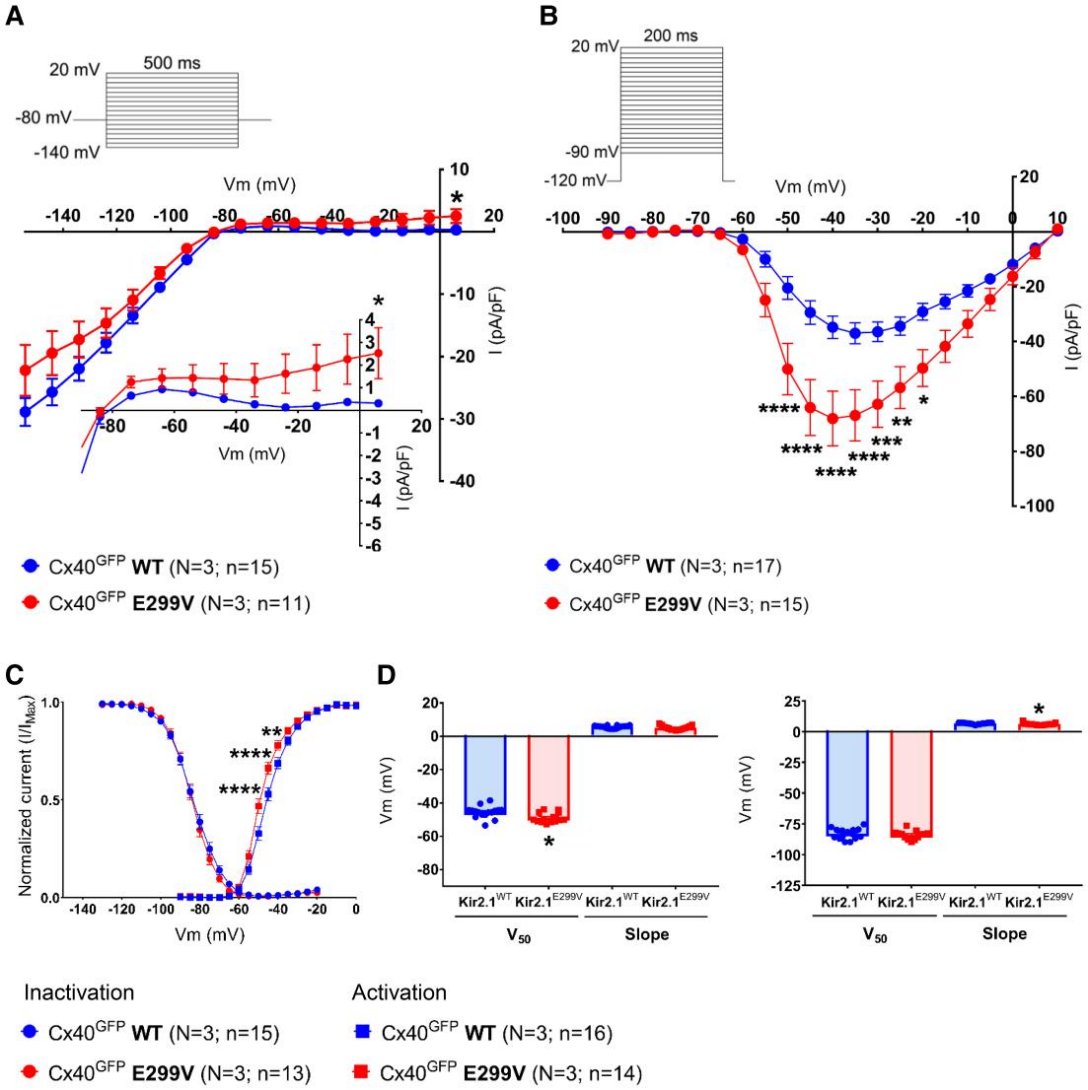
Kir2.1^E299V^ increases I_Na_ density in cardiac Purkinje cardiomyocytes. *A*, I_K1_ IV relationships for Cx40^GFP^ AAV-Kir2.1^WT^ (*N* = 3, *n* = 15) and Cx40^GFP^ AAV-Kir2.1^E299V^ (*N* = 3, *n* = 11) Purkinje cells (*P = 0.0163 at +6 mV). *B*, Superimposed I_Na_ IV relationships (*N* = 3 and *n* = 15–17; ****P < 0.0001, ***P < 0.001, **P < 0.01, and *P < 0.05 when indicated). *C*, Sodium activation and inactivation curves (*N* = 3, *n* = 13–16; ****P < 0.0001 and **P < 0.01). *D*, Graphs show sodium activation (left panel) and inactivation (right panel) parameters (V_50_ and slope) (*N* = 3; *n* = 13–16, *P < 0.05). Two-way ANOVA (IV and sodium activation/inactivation curves) and Mann–Whitney test (activation/inactivation parameters) applied for comparisons.

The slight abbreviation in the QRS complex duration in Kir2.1^E299V^ mice suggested a confirmed increase in the ventricular CV. Then, we wanted to know the reason for the PR interval shortening in mutant animals. We infected transgenic Cx40^GFP^ with AAV-Kir2.1^WT^ and AAV-Kir2.1^E299V^ to localize and isolate infected Purkinje cardiomyocytes and determine changes in I_K1_ and I_Na_.^33,69–72^ I_K1_ recordings showed a small but significant reduction of inward-going rectification in Cx40^GFP^-Kir2.1^E299V^ compared to control Cx40^GFP^-Kir2.1^WT^ cells (*Figure 8A*). However, a more remarkable difference occurred at the cardiac sodium current level: Cx40^GFP^-Kir2.1^E299V^ cells manifested a significantly higher I_Na_ density than Cx40^GFP^-Kir2.1^WT^ cells, and a shift of I_Na_ activation to negative voltages (*Figure 8B–D*). Together, these results clearly explain the shortening of the PR interval and the slightly abbreviated QRS complex of mutant mice. They contribute to a higher CV conferring protection against ventricular arrhythmias in Kir2.1^E299V^ mice, and explaining their chamber-specific inducibility.

## 4. Discussion

The most important results of this original study are as follows: (i) We have generated the first *in-vivo* model of SQTS3 able to reproduce the phenotypical electrical characteristics of a patient with the Kir2.1^E299V^ mutation.^14^ (ii) On ECG, the QT interval of Kir2.1^E299V^ mice was significantly shorter than Kir2.1^WT^ mice. (iii) Arrhythmia inducibility was greater in the atria in than the ventricles of Kir2.1^E299V^ mice. (iv) Both atrial and ventricular Kir2.1^E299V^ cardiomyocytes generated extremely abbreviated APs due to lack of inward-going rectification. (v) There were significant chamber-specific effects of the Kir2.1^E299V^ mutation at the ion channel level: while in Kir2.1^E299V^ atrial cardiomyocytes I_K1_ presented a reduced slope conductance, in ventricular cardiomyocytes the mutation increased cell excitability by shifting I_Na_ activation and steady-state inactivation in the hyperpolarizing direction, which protected the ventricles against arrhythmia induction. In addition, in the cardiac conduction system cells, the mutation caused a higher I_Na_ density contributing to increase the CV. Therefore, in the ventricles, the Kir2.1^E299V^ mutation reduces Kir2.1 current rectification to shorten the APD and increases excitability by modifying Na_V_1.5 function. (vi) In atrial cardiomyocytes, while the mutation increased the outward I_K1_ at positive voltages, a greater proportion of Kir2.1^E299V^-Kir2.2^WT^ channels impaired polyamine block in the presence of a reduced pore diameter. Altogether, the results provide novel functional interactions between Kir2.1 and Na_V_1.5 channels and insight into the mechanism underlying greater atrial than ventricular arrhythmogenesis in the mouse model and a patient with SQTS3 due to the Kir2.1^E299V^ mutation.

We used AAV9 technology^26^ to generate the first SQTS3 mouse model with gene constructs containing Kir2.1^E299V^ vs. the Kir2.1^WT^ version as control. The cardiac mechanisms are too complex to investigate them in heterologous expression systems, and here we provide an *in-vivo* model that changes the landscape for the study of hereditary arrhythmias associated with SQTS3. Introduction of exogenous human Kir2.1^E299V^ in mice expressing the murine Kir2.1^WT^ endogenously reproduces a heterozygous condition where the dominant negative effect of the mutation mimics the patient’s genetic environment in the heart. Importantly, infection using the human WT or mutant Kir2.1 sequences did not alter the endogenous expression of Kir2.x subtypes or caused Kir2.1 overexpression in the cardiac tissue.

Mutant mice presented significant QT abbreviation and increased susceptibility to arrhythmias. Programmed electrical stimulation (PES) in the right atria, His bundle or RV of Kir2.1^WT^ and Kir2.1^E299V^ groups demonstrated that, by far, the largest proportion of mutant mice responded with supraventricular arrhythmias like AF. Also, the longest-lasting events were recorded in the atria. At the ionic level, Kir2.1^E299V^ ventricular cardiomyocytes revealed a gain-of-function in the outward I_K1_ due to a lack of inward-going rectification. This behaviour was similar in mutant atrial cardiomyocytes, which, unlike the ventricles, also had reduced I_K1_ at potentials negative to −80 mV. *In-silico* structural modelling and inside-out patch-clamp experiments confirmed the lack of rectification in Kir2.1^E299V^ cardiomyocytes underlying the gain-of-function. As glutamic acid was replaced by valine, the mutation induced a loss of negative charges in the channel pore. Consequently, positively charged polyamines failed to penetrate sufficiently to block mutant Kir2.1^E299V^ channels.

There is increasing evidence that channels function as part of macromolecular complexes and interact with other proteins, including ion channels.^21–25,34,36,73,74^ Evidence also indicates that mutations in ionic channels or in their regulatory subunits cause modifications in the functional, structural, or kinetic properties of many other interactors.^23,24,34^ Some of them are important in maintaining excitability and excitation–contraction coupling, so punctual genetic alterations in apparently unrelated proteins provoke unexpected changes in other cell functions. Thus, we should not treat channelopathies as monogenic diseases, since even though they may be caused by specific mutations in one gene, a given mutation also affects the products of other genes. Such a premise was borne out in the *in-vivo* SQTS3 model, which allowed the discovery that the Kir2.1^E299V^ mutation modified the biophysical properties of the I_Na_ in ventricular cardiomyocytes and its density in cardiac Purkinje cells. In mutant ventricular cardiomyocytes, Nav1.5 activates at more negative voltages than WT and its inactivation is also left-shifted, which likely contributed to increasing I_Na_ availability at potentials between −80 and −60 mV.

The speed of excitation of a cardiomyocyte in response to an external stimulus is determined by the rate of approach to threshold (foot potential) and the maximum rate of depolarization (dV/dt_max_) during phase 0 of the AP (see Supplementary material online, *Figure S19A*). The foot potential depends on the balance between the magnitude of inward current provided by the stimulus, the time needed to charge the membrane capacitance and the amount of outward I_K1_ opposing the depolarization. Once threshold has been reached, dV/dt_max_ will depend on the number of sodium channels available for excitation.^75^ Similarly, the velocity of impulse propagation in the cardiac syncytium will depend on the amount of charge carried by the AP of a given cardiomyocyte to excite downstream neighbours, the time needed to charge their membrane capacitance, the amount of outward I_K1_ opposing depolarization and the availability of sodium channels for excitation.^76^ Thus, to determine why CV increased in the mutant ventricles with respect to WT, in cardiomyocytes we compared the amount of current needed to reach threshold as the relation between sodium channel availability and the charge needed for excitation, normalized by the access resistance (see Supplementary material online, *Figure S19B*). Therefore, despite the absence of dV/dt_max_ change, the more negative threshold potential and lower current needed for excitation likely enabled for a shorter foot of the conducted AP, accelerating conduction in the mutant ventricles, which accounted for the slightly shorter QRS interval. On the other hand, the increased I_Na_ density in the mutant Purkinje fiber network was likely responsible for the shortening of the PR interval. Hence, unexpectedly, the same mutation differentially affected each of the cardiac chamber and cell type, which explains the atrial arrhythmia predisposition and absence of ventricular arrhythmias in the mouse and the patient with the Kir2.1^E299V^ mutation. Most likely, specific Kir2.1 interactors in each of the cardiac regions modify the electrical properties differently, which should explain the dissimilar outcomes with the same mutation.

Kir2.1 and Na_V_1.5 traffic together from the sarcoplasmic reticulum and they interact with multiple proteins capable of modifying their targeting and distribution.^21–25,34,36,73,74^ Proteins like SAP97 and α1-Syntrophin act as scaffolds that keep Kir2.1 and Na_V_1.5 together at the membrane through their PDZ (Postsynaptic density protein, *Drosophila* disc large tumour suppressor, and Zonula occludens-1 protein) binding domains. *In-vitro* experiments have demonstrated that inhibition or absence of these interacting proteins causes alterations in both I_K1_ and I_Na_.^20,22,77–81^ However, in ventricular cardiomyocytes expressing Kir2.1^E299V^, we have not seen changes in the distribution or levels of either channel or their specific interactors, which rule out the above proteins as mediators of the arrhythmogenic consequences of the mutation.

Some of the Na_V_1.5 β subunits are known to predictably modify I_Na_ activation and inactivation^35,62–68^ in cardiac and other excitable cells, and they also interact with other channels. Hu *et al*. demonstrated that a variant in *SCN1Bb* gene encoding the β1 subunit caused alterations in I_Na_ and I_to_, as β1 has functional and structural association with both Na_V_1.5 and K_V_4.3.^82^ These regulatory subunits would be interacting with other apparently unrelated channels and taking part of the same macromolecular complexes. Na_V_1.5 β2 and β4 are expressed mainly in the ventricles.^68^ Apparently, Na_V_β2 alone does not have effects on the kinetic properties of Na_V_1.5,^35^ but Na_V_β4 expression would be able to change I_Na_ biophysical parameters.^62^ However, transfection with Kir2.1^E299V^ along with β4 in the HEK-Na_V_1.5 cell line failed to reproduce the results seen in mutant ventricular cardiomyocytes (see Supplementary material online, *Figure S18*). The question remains why the Kir2.1^E299V^ mutation leads to such an unexpected gain-of-function modification in Na_V_1.5 so specifically in ventricular but not atrial cardiomyocytes. Answering this question will require exploration of yet unidentified but possible Kir2.1 interactions with Na_V_1.5 partner proteins.

The atria have certain characteristics that may contribute to arrhythmogenesis in light of APD shortening induced by I_K1_ gain-of-function. The complex anatomy of the atria is potentially arrhythmogenic due in part to the highly intricate arrangement of the endocardial pectinate muscles, which may facilitate both inducibility and maintenance of AF.^83^ Computational and experimental studies have demonstrated that the pectinate muscles contribute stabilizing re-entry and fibrillatory conduction.^84^ Moreover, the spatially heterogeneous ion channel distribution and electrical properties throughout both atria also make them particularly susceptible to arrhythmias due to I_K1_ gain-of-function,^85^ even in the absence of other electrophysiological alterations.

Panama *et al*. showed that both Kir2.x expression and I_K1_ properties are spatially heterogeneous in the mouse heart.^59^ Gaborit *et al*. demonstrated that the ventricles express higher levels of Kir2.1, but the expression profile of Kir2.2 was the same in both cardiac chambers.^86^ Here, we confirmed that the ventricles have higher levels of Kir2.1 than the atria, a pattern that remains even in the presence of the E299V mutation (*Figure 6*). Seeing slightly higher levels of Kir2.2 in murine atria than ventricles, we assumed that in the atrium there is a larger number of Kir2.x channels containing Kir2.2 subunits compared with the ventricles. I_K1_ generated by dimers expressing Kir2.1^E299V^-Kir2.2^WT^ and transfected into HEK-293 T cells was similar to atrial cardiomyocytes. In addition, heterotetrameric Kir2.1^E299V^-Kir2.2^WT^ channels simulated *in-silico* had a reduced pore diameter that could explain their reduced conductance at voltages negative to −80 mV. We, therefore, propose that, together with the complex cardiac structure, such a low Kir2.1 pore conductivity underlies the greater atrial than ventricular arrhythmogenic potential in the Kir2.1^E299V^ mice.

The young patient in whom we discovered the Kir2.1^E299V^ mutation presented an extremely short QTc interval and AF. Such a phenotype provides validation to our results, as the mouse recapitulated the most important aspects of the patient’s electrical phenotype. However, extrapolation of our results to the clinic and the patient with SQTS3 should be done with extreme caution. After all, Kir2.3 is the most predominant Kir2.x subtype in the human atrium,^86^ and it does not interact reciprocally with Na_V_1.5 channels.^22^ We, therefore, conducted additional *in silico* studies using Kir2.1^E299V^-Kir2.3^WT^ heterotetramers (see Supplementary material online, *Figure S20*). The simulated channels had defects in the pore and its polarity, and were unable to bind polyamines, indicating they would not rectify either in human atrial cardiomyocytes. Therefore, even without any modifications in the voltage dependence and biophysical properties of Na_V_1.5, the absence of I_K1_ rectification and reduced pore conductance caused by the Kir2.1^E299V^ mutation in atrial cardiomyocytes would be sufficient to underlie the initiation and maintenance of AF in the heterogeneous atria of a SQTS3 patient.

Additionally, our results stablish the molecular basis of SQTS3 and open new strategic lines for the development of drugs based on the polyamines’ skeleton. These positively charged molecules present a high capacity for blocking Kir2.1 and could be chemically modified for reducing the hyperfunctionality of Kir2.1^E299V^ mutant channels. These new therapies may help to preventing life-threating arrhythmias and SCD in SQTS3 and possibly other diseases.

In conclusion, this work contributes to unravel the arrhythmogenic consequences of the gain-of-function mutation Kir2.1^E299V^ causing SQTS3. Knowing the molecular and electrical environment that triggers lethal arrhythmias in patients suffering from this syndrome may lead to develop diagnostic tools and new therapeutic strategies to reduce their morbidity and mortality. Moreover, unravelling the molecular mechanisms underlying rare and lethal syndromes, such as SQTS3, contributes to increase knowledge that can be applied to the management of other more prevalent cardiac diseases.

### 4.1 Limitations

We have used mice to investigate arrhythmogenic mechanisms in SQTS3, but we are well aware of the potential limitations of this animal model to study a human disease. Heart rate and repolarization features between mice and humans are different, as they are governed by different sets of potassium currents, a fact that alters the AP and QT interval durations. For example, mice do not present I_Kr_ or I_Ks_, the rapid and slow delayed rectifier currents. This sense, while chloroquine and flecainide are known to inhibit not only I_K1_, but I_Kr_ as well, the latter effect would not be reflected in mouse QT data. Structurally, there are also remarkable differences between the murine and human hearts, such as the heart size. Therefore, results about specific mechanisms of arrhythmia in the mice should not be extrapolated directly to the clinic, and more studies using appropriate preclinical models would be needed to ensure rigorous translation of our results to the human patient. Moreover, regarding the AAVs, although AAV vectors delivered to the heart show a high rate of infection (over 95%) and genetic load (over 60% of the cardiomyocytes have between one and three copies of the transgene), this system always generates a mosaic cellular distribution of Kir2.1^WT^ and Kir2.1^E299V^ expression in the heart. Yet, despite the above limitations, we stand by our results, which provide novel insights into the mechanisms of differential chamber-specific electrical remodelling underlying atrial arrhythmogenesis and ventricular protection in a mouse model of SQTS3.

## Supplementary Material

cvae019_Supplementary_Data

## Data Availability

The data underlying this article are available in the article and in its online supplementary material. Additional data will be shared on request to the corresponding authors.

## Notes

Translational perspective The pathologic genetic variation of the strong inward rectifier K^+^ channel (Kir2.1^E299V^) leads to SQTS3 with atrial-specific arrhythmias while paradoxically enhancing Purkinje fiber excitability and ventricular conduction velocity. The availability of an animal model of SQTS3 may lead to the identification of new molecular targets in the design of novel drugs to treat a cardiac disease that currently has no defined therapy. The chemical skeleton of polyamines, which block Kir2.1 channels at specific voltages, may serve as a template for designing new drugs capable of correcting hyperfunctional Kir2.1 channels, and preventing arrhythmias in SQTS3 and possibly other diseases.
