# Supplementary material for: The Kir2.1E299V mutation increases atrial fibrillation vulnerability while protecting the ventricles against arrhythmias in a mouse model of short QT syndrome type 3
Source: Cardiovasc Res. 2024 Jan 23;120(5):490–505. doi: 10.1093/cvr/cvae019 (PMC11060485; doi:10.1093/cvr/cvae019)
Supplement: cvae019_Supplementary_Data [file cvae019_supplementary_data.docx]

**SUPPLEMENTARY MATERIAL**

**The Kir2.1^E299V^ mutation increases atrial fibrillation vulnerability while protecting the ventricles against arrhythmias in a mouse model of Short QT Syndrome type 3**

Ana I Moreno-Manuel^1^, MSc, Álvaro Macías^1^ *, PhD, Francisco M Cruz^1^ *, PhD, Lilian K Gutiérrez^1^, MSc, , Fernando Martínez^1,2^, PhD, Andrés González-Guerra^1,#^, Isabel Martínez Carrascoso^1^, MSc, Francisco José Bermúdez-Jimenez^1,3^, MD PhD, Patricia Sánchez-Pérez^1^, PhD, María Linarejos Vera-Pedrosa^1^, MSc, Juan Manuel Ruiz^1^, MSc, Juan A Bernal^1,2^ *, PhD, José Jalife^1,2,4^, MD PhD

1. Centro Nacional de Investigaciones Cardiovasculares (CNIC), 28029, Madrid, Spain.
2. CIBER de Enfermedades Cardiovasculares (CIBERCV), Madrid, Spain.
3. Hospital Universitario Virgen de las Nieves, 18014, Granada, Spain.
4. Departments of Internal Medicine and Molecular and Integrative Physiology, University of Michigan, 48109, Ann Arbor, MI, USA.

^#^ Current address: European Molecular Biology Laboratory (EMBL), 00015, Monterotondo, Lazio, Italy.

^*^ Corresponding authors:

Juan Antonio Bernal, PhD

Viral Vector Unit (ViVU)

Centro Nacional de Investigaciones Cardiovasculares Carlos III

Melchor Fernández Almagro 3, 28029, Madrid, Spain

Email: [jabernal@cnic.es](mailto:jabernal@cnic.es)

Telephone: +34-91 453 12 00 (Ext. 3307) / FAX: +34-91 453 12 65

Álvaro Macías Martínez, PhD

Cardiac Arrhythmia Laboratory

Centro Nacional de Investigaciones Cardiovasculares Carlos III

Melchor Fernández Almagro 3, 28029, Madrid, Spain

Email: [alvaro.macias@cnic.es](mailto:alvaro.macias@cnic.es)

Telephone: +34-91 453 12 00 (Ext. 4311) / FAX: +34-91 453 12 65

Francisco Miguel Cruz, PhD

Cardiac Arrhythmia Laboratory

Centro Nacional de Investigaciones Cardiovasculares Carlos III

Melchor Fernández Almagro 3, 28029, Madrid, Spain

Email: fmcruz@cnic.es

Telephone: +34-91 453 12 00 (Ext. 4308) / FAX: +34-91 453 12 65

**Short title:** Arrhythmogenic mechanisms underlying SQTS3.

**SUPPLEMENTARY FIGURES**


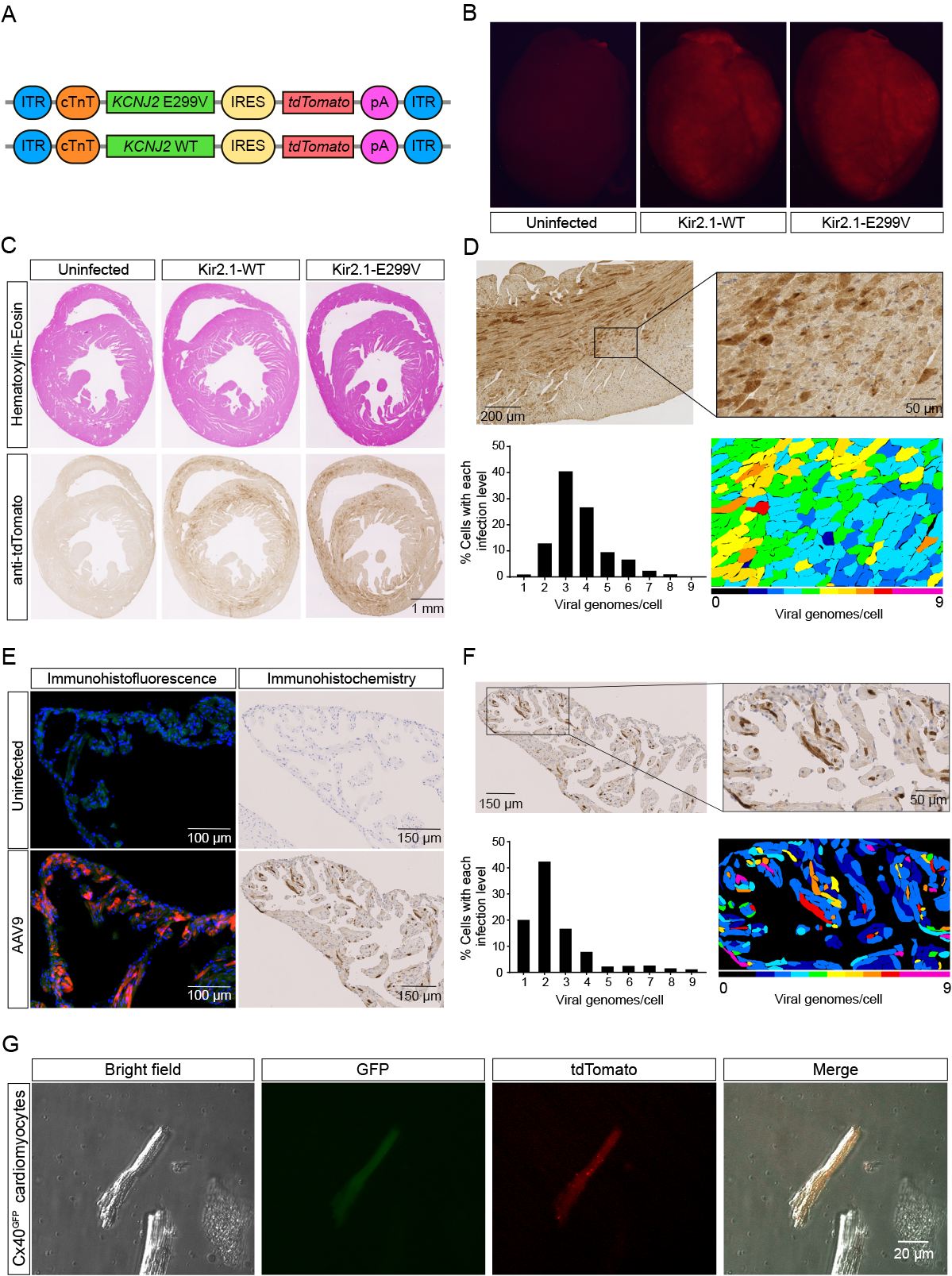


**Supplementary Figure 1. The use of adeno-associated virus serotype 9 (AAV9) to generate mouse models results in the infection of ventricles, atria and cardiac conduction system cells. A**, AAV9 constructs containing the cardiac troponin-T promoter (cTnT), the *KCNJ2* (mutant or WT) gene, the *Tomato* reporter gene*,* an internal ribosome entry site (IRES) and the poly-adenine (pA) sequence, all flanked by inverted terminal repeats (ITRs). **B**, Representative fluorescence images of uninfected, Kir2.1^WT^ and Kir2.1^E299V^ hearts (3 hearts per group). **C**, **Top**, Haematoxylin-Eosin staining shows normal structure in infected Kir2.1^WT^ and Kir2.1^E299V^ hearts regarding uninfected ones. **Bottom**, tdTomato immunostaining of uninfected, Kir2.1^WT^ and Kir2.1^E299V^ hearts. **D**, Immunohistochemistry of tdTomato confirmed infection in Kir2.1^WT^ and Kir2.1^E299V^ hearts. Note that the infection level was ~95%, with >50% cardiomyocytes expressing 2-4 viral genomes/cell. **E**, tdTomato immunohistofluorescence/chemistry of uninfected and AAV9 atria show specific staining in AAV9 tissues. **F**, Quantification of the viral genomes/atrial cell in AAV9 tissues. **G**, Cardiac conduction system cells positive for Cx40^GFP^ and infected with AAV9 show tdTomato expression.

.


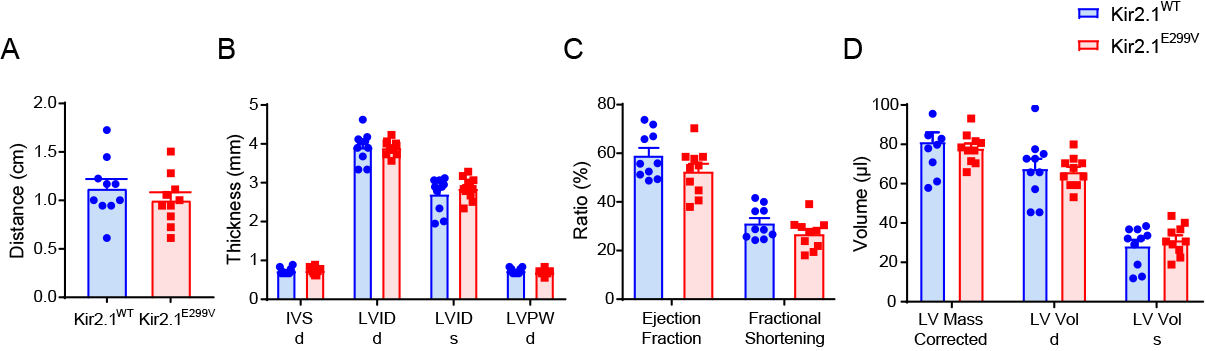


**Supplementary Figure 2. Echocardiographic analysis of Kir2.1^WT^ and Kir2.1^E299V^ mice reveals normal cardiac structure and function. A**, Tricuspid Annular Plane Systolic Excursion (TAPSE) measurements for Kir2.1^WT^ (blue) and Kir2.1^E299V^ (red) shows no defects in right ventricular function. **B**, Interventricular septal end diastole (IVSd); left ventricular internal diameter end diastole and end systole (LVIDd and LVIDs); left ventricular posterior wall end diastole (LVPWd). **C**, Ejection Fraction (EF) and Fractional Shortening (FS) have the same values in both groups. **D**, Corrected left ventricular mass (LV Mass C); left ventricular volume in diastole and in systole (LV Vol d and LV vol s measured in µl) did not reveal any alteration. We used 10 animals per condition. Unpaired 2-tailed Student’s t-test was applied.


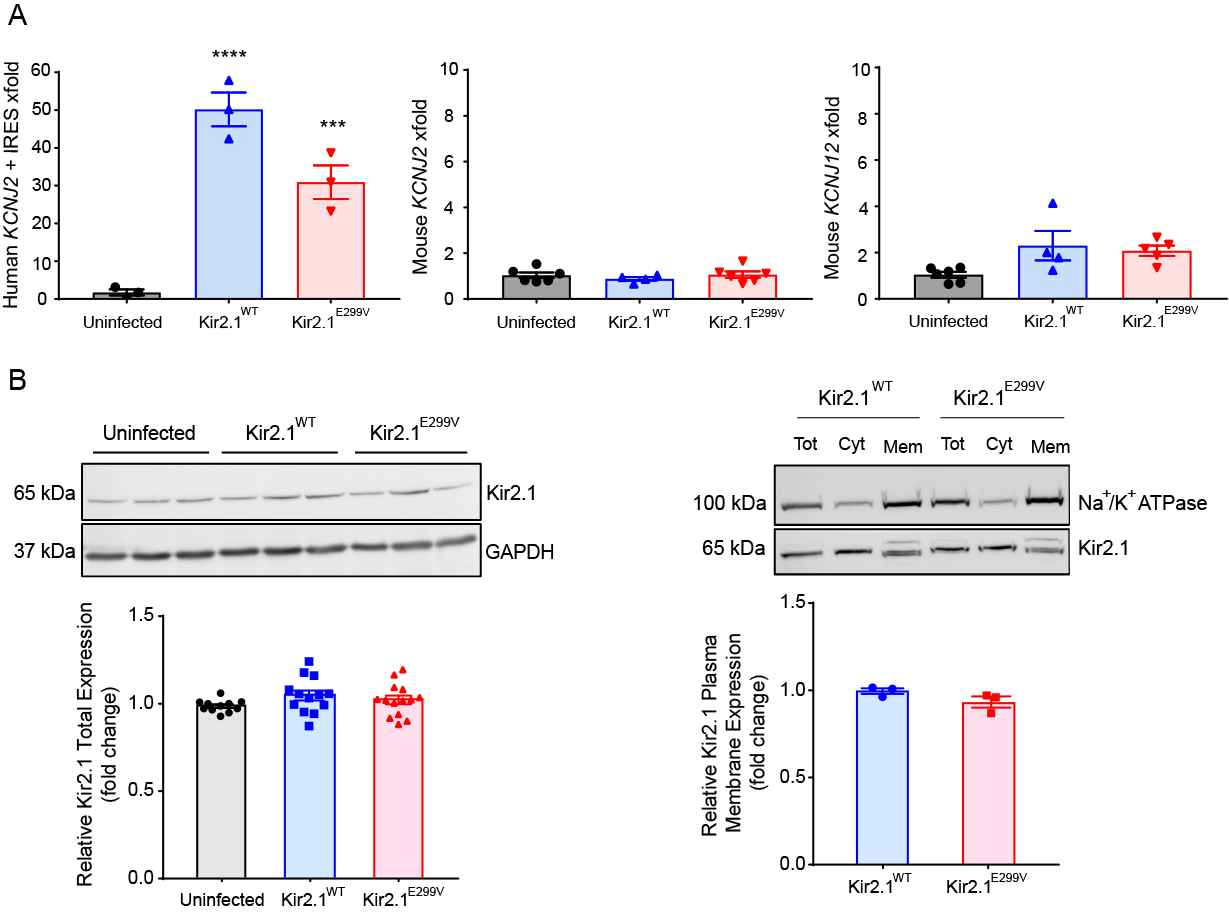


**Supplementary Figure 3. AAV9 generated mouse models express human Kir2.1^WT^ or Kir2.1^E299V^ without affecting endogenous Kir2.x levels.** **A**, Left, Analysis of expression of human *KCNJ2* (Kir2.1) transcripts by qRT-PCR in uninfected, Kir2.1^WT^ and Kir2.1^E299V^ hearts (N=3 different hearts per condition with 3 replicates per heart; ***p<0.001 when comparing Kir2.1^WT^ with uninfected mice; **p=0.009 when comparing Kir2.1^E299V^ *vs* uninfected). Middle and right, Expression levels of endogenous *KCNJ2* (Kir2.1) and *KCNJ12* (Kir2.2) levels in the same experimental groups (N=4-6 animals per group). **B**, Left, Kir2.1 total protein levels in uninfected, Kir2.1^WT^ and Kir2.1^E299V^ hearts (N=11-14 animals per condition). Right, Kir2.1 membrane protein levels in Kir2.1^WT^ and Kir2.1^E299V^ hearts (N=3 animals per condition). One-way ANOVA (qRT-PCR and Kir2.1 total protein data) and Mann-Whitney test (Kir2.1 membrane) were applied for statistics.


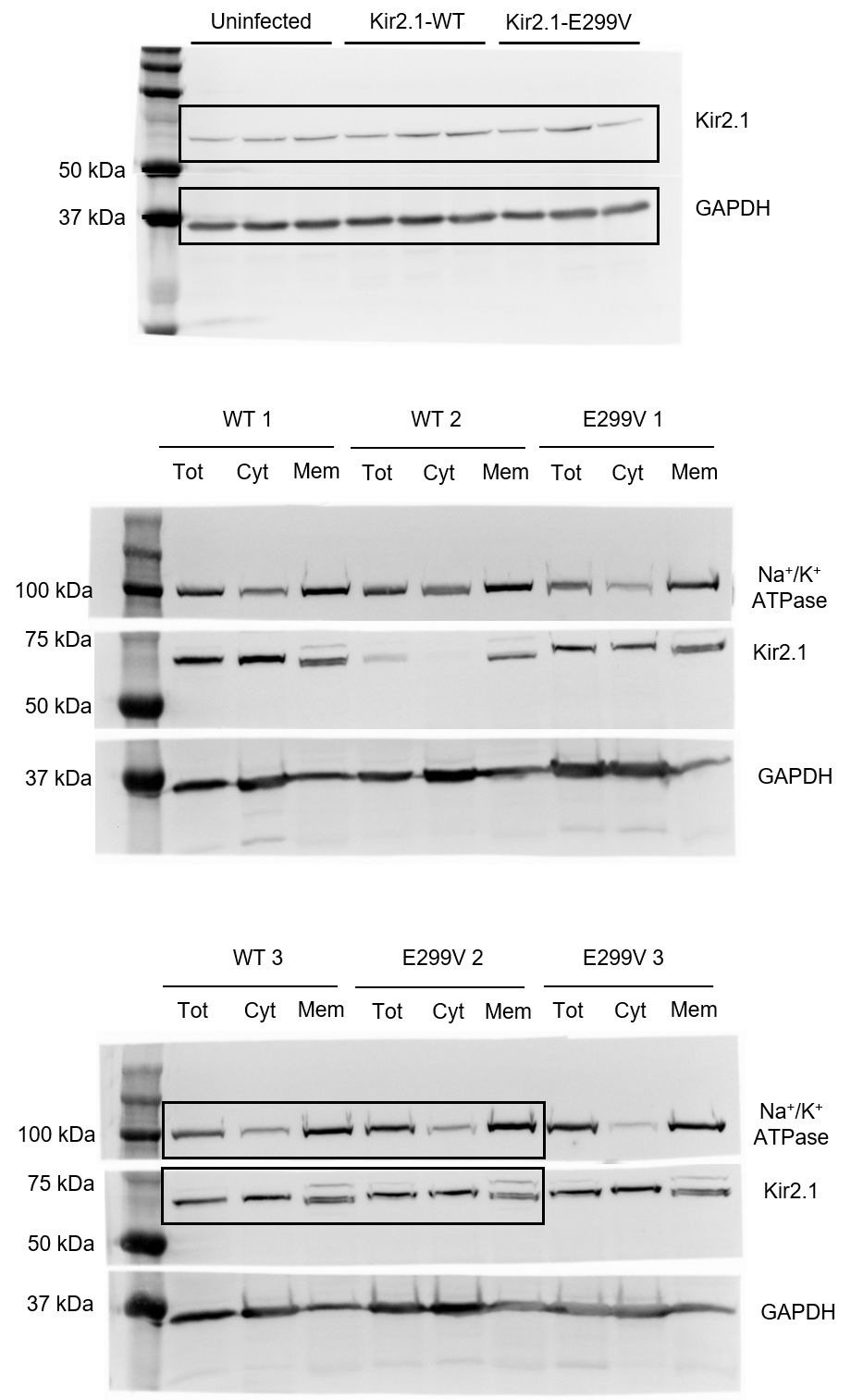


**Supplementary Figure 4.** Uncropped images for western blot gels presented in Supplementary Figure 1. Black boxes mark the borders of the final cropped images.

**
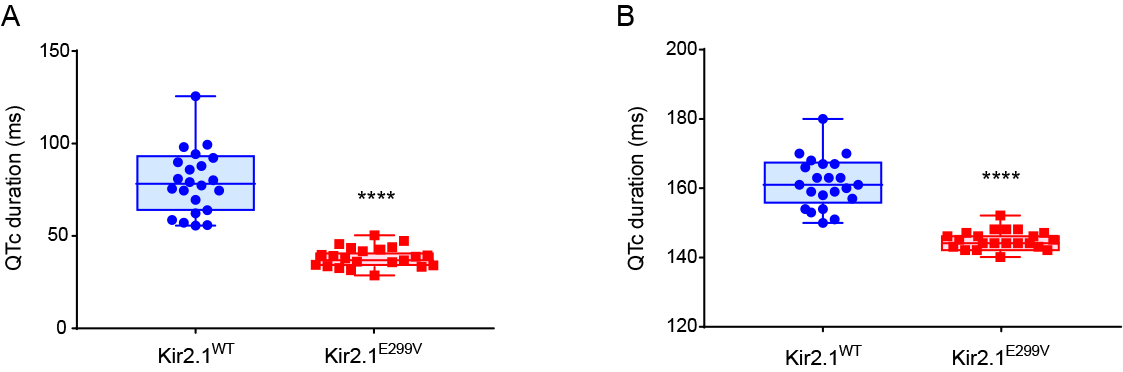
**

**Supplementary Figure 5. The Kir2.1^E299V^ mouse model presents an abbreviated QT corrected (QTc) interval.** **A**, QTc intervals of Kir2.1^WT^ and Kir2.1^E299V^ mice (80.63±3.54ms *vs* 38.94±0.99ms, respectively; N=28-30, ****p<0.0001) using the Bazett’s equation. **B**, QTc intervals of Kir2.1^WT^ and Kir2.1^E299V^ animals (162.30±1.44ms *vs* 145.50±0.50ms, respectively; N=28-30, ****p<0.0001) applying the Framingham’s correction. We used the unpaired 2-tailed Students’ t-test for comparisons.


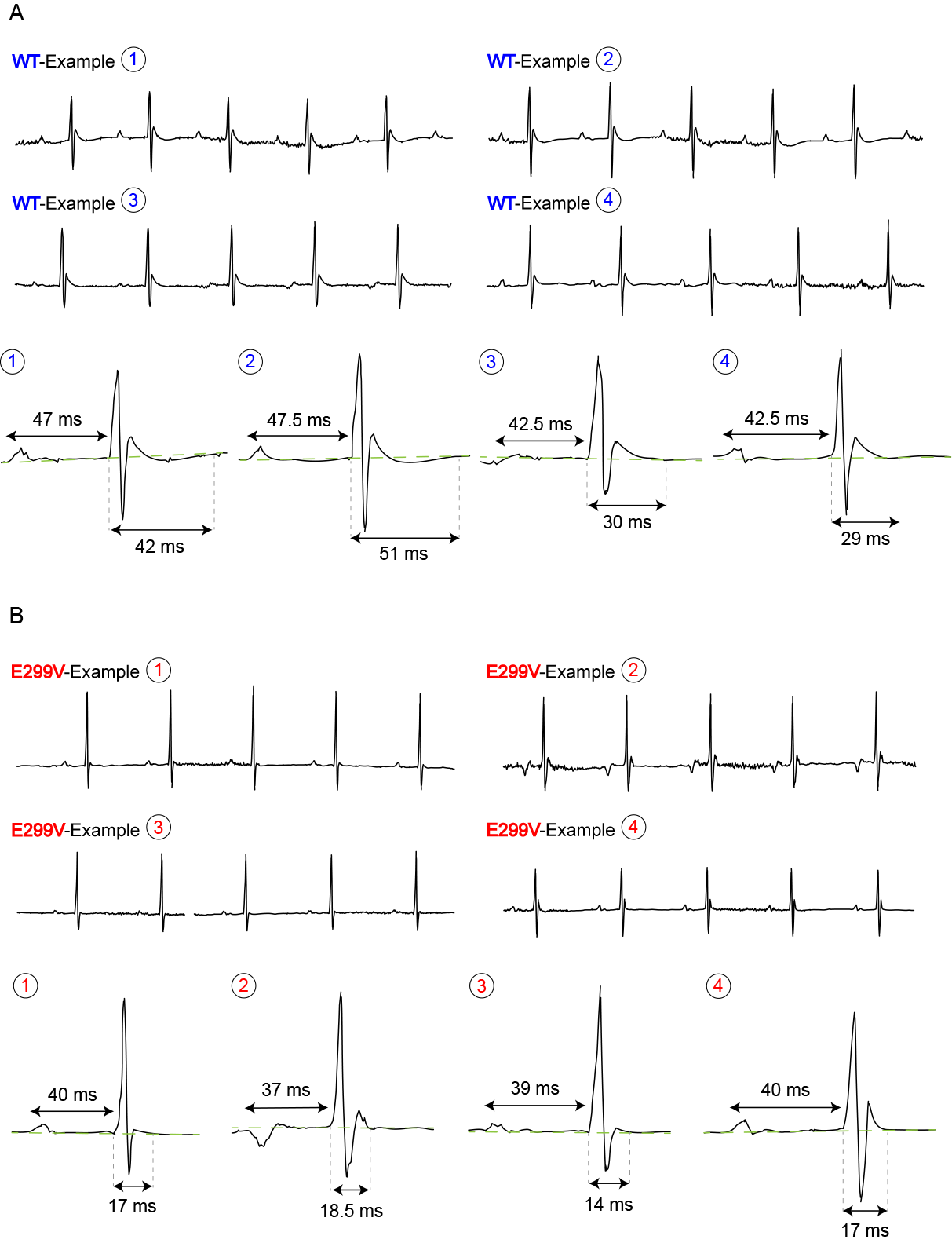


**Supplementary Figure 6. Kir2.1^E299V^ mutation abbreviates the QT interval duration. A**, Sample Lead-II ECGs from four different Kir2.1^WT^ mice under basal conditions. **B**, Sample ECGs from four independent Kir2.1^E299V^ animals under basal conditions. The insets correspond to each example (1-4 for each genotype). PR and QT interval durations are indicated.

**
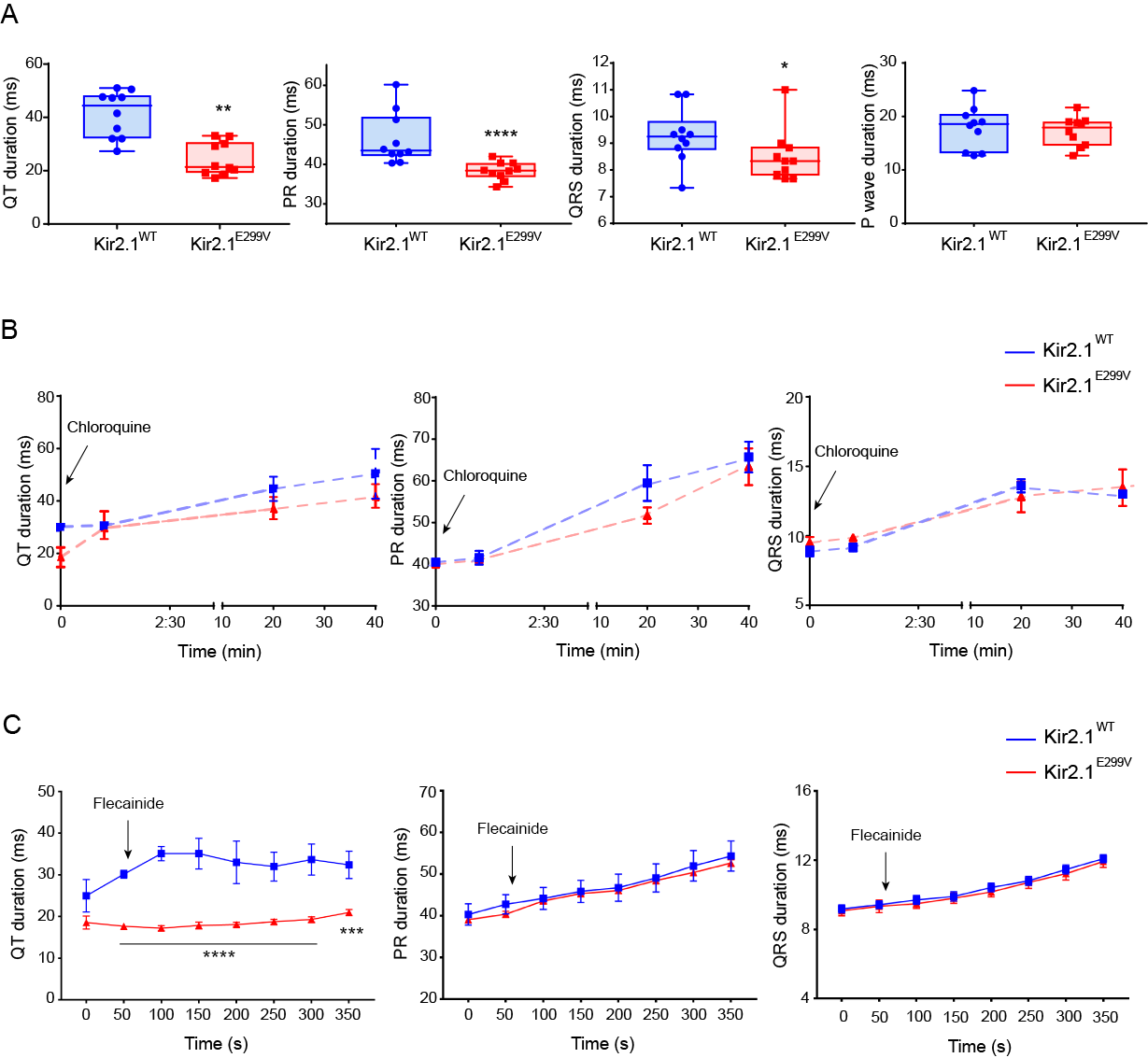
**

**Supplementary Figure 7. Drug testing shows lack of efficacy of possible treatments for SQTS3. A**, Analysis of the isoproterenol (ISO) 5mg/kg i.p. effect on QT (**p<0.01; N=10), PR interval (****p<0.0001; N=10), QRS complex (*p=0.0059; N=10) and P wave durations (p>0.5; N=10) in Kir2.1^E299V^ (red) compared to Kir2.1^WT^ mice. **B**, Temporal effects of chloroquine 40mg/kg i.p. on the electrocardiogram (ECG). QT, PR and QRS durations are indicated (p>0.5 at all times tested; N=3). **C**, Temporal effects of flecainide 10mg/kg i.p. on ECG. The QT of the Kir2.1^WT^ mice was prolonged by the drug but had no effect on Kir2.1^E299V^ mice (p<0.05 for all registration times; N=5-10). Unpaired 2-tailed Student’s t-test (panel A, ISO QT and ISO P wave), Mann-Whitney test (panel B, ISO PR and ISO QRS) and Two-way ANOVA (panels B and C) for comparisons.


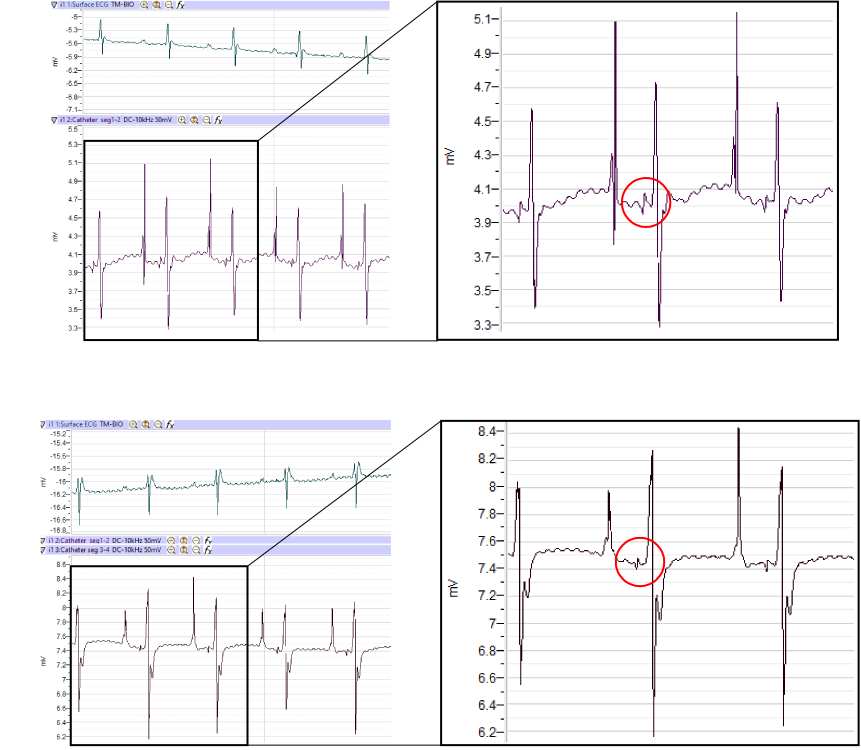


**Supplementary Figure 8. His bundle signal detected between the atrial and ventricular deflections in the intracardiac electrogram recordings of our programmed electrical stimulation (PES) experiments.** In these two examples obtained from two different animals, the crops show the surface ECG on top, and the catheter signal below. On the enlarged inserts, we have enclosed the His bundle signal with a red circle.


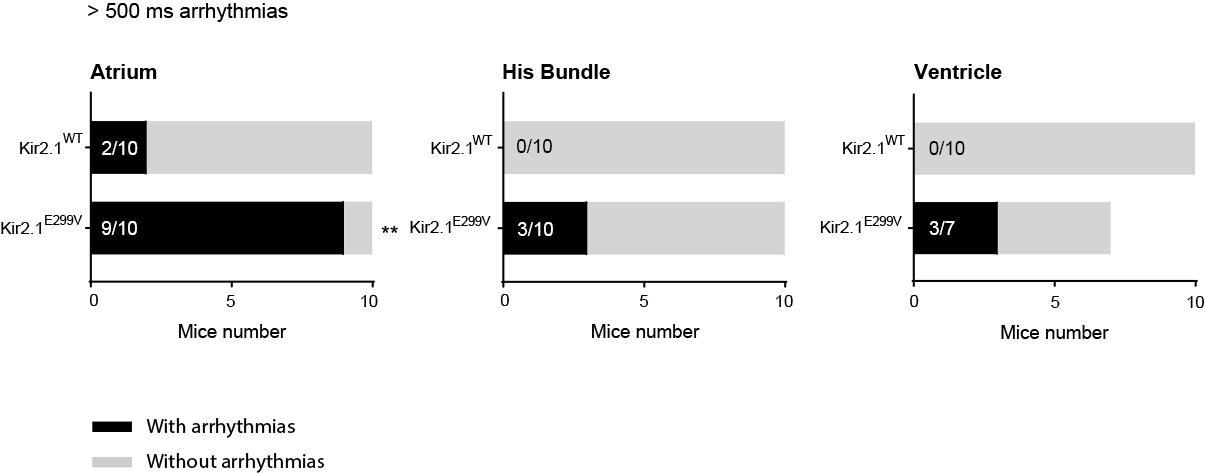


**Supplementary Figure 9. Predominance of atrial arrhythmias by intracardiac stimulation in Kir2.1^E299V^ mice.** Arrhythmias of >500ms duration in Kir2.1^WT^ and Kir2.1^E299V^ mice induced by stimulating right atrium (**p=0.0055; N=10), His bundle (p=0.2105; N=10) or right ventricle (p=0.0515; N=7-10). Fisher’s exact test for contingency data presenting the presence or absence of arrhythmias.

**
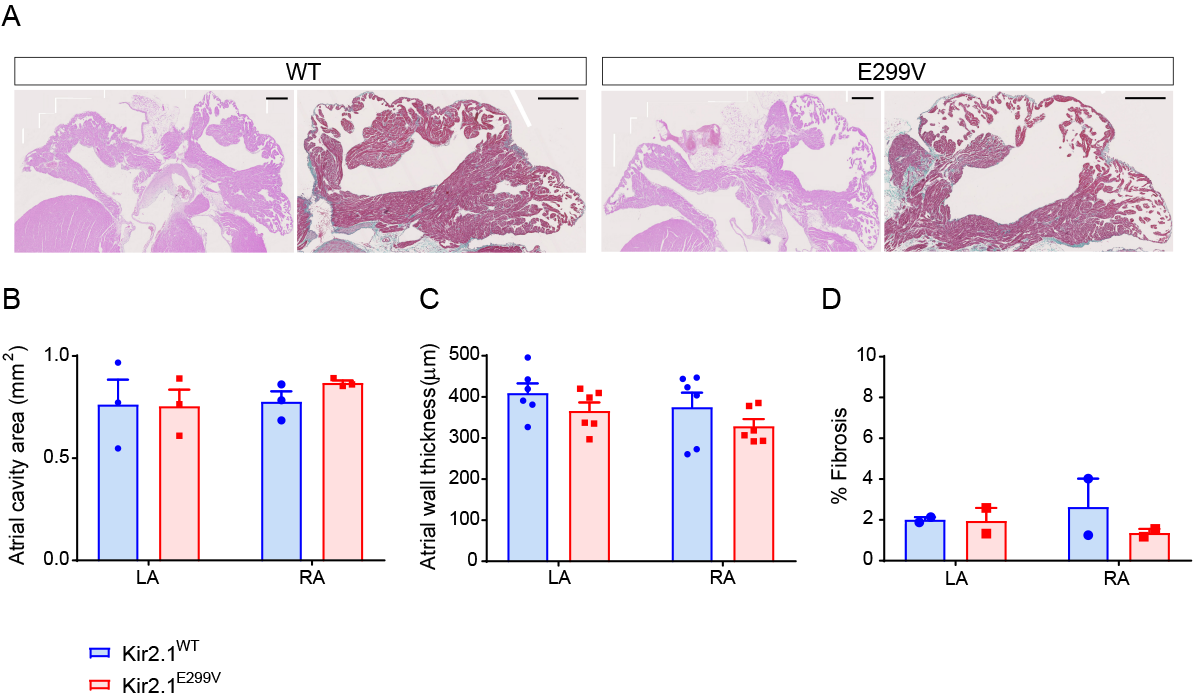
**

**Supplementary Figure 10. The atria of Kir2.1^E299V^ mice do not present any structural alteration.** **A**, Haematoxylin-Eosin staining of Kir2.1^WT^ and Kir2.1^E299V^ atria is shown on the left part of each panel, and Masson's Trichrome staining is shown on the right. Scale bars, 400µm. **B**, Measurements of atrial dilation (mm^2^) in the left (LA) and right atria (RA) of Kir2.1^WT^ (blue) and Kir2.1^E299V^ (red) mice (N=3, p>0.999 for LA and p=0.3 for RA). **C**, Atrial wall thickness measured in two regions of each atrium per animal (N=3, p=0.4 for both LA and RA). D, Percentage of fibrosis in LA and RA of Kir2.1^WT^ (blue) and Kir2.1^E299V^ (red) mice (N=2, p>0.999 for LA and p=0.6667 for RA).


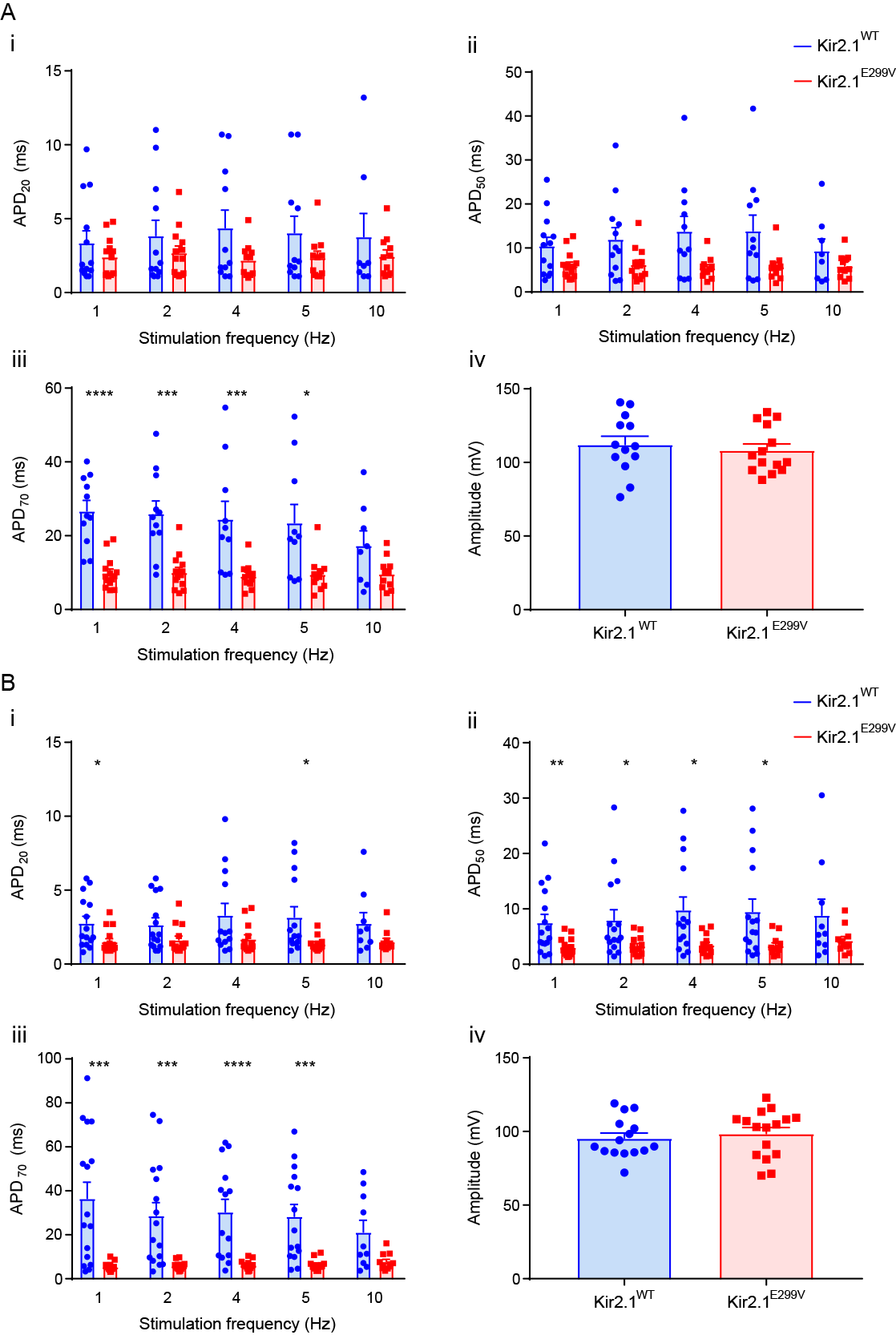


**Supplementary Figure 11. Kir2.1^E299V^ shortens the APD in atrial and ventricular cardiomyocytes. A**, Atrial cardiomyocytes. Kir2.1^WT^ (blue; N=3, n=8-13) and Kir2.1^E299V^ (red; N=3, n=10-14). **i**, APD at 20% repolarization for 1, 2, 4, 5 and 10Hz (p>0.05). **ii**, APD at 50% repolarization for 1, 2, 4, 5 and 10Hz (p>0.05). **iii**, APD at 70% repolarization for 1, 2, 4, 5 and 10Hz (****p<0.0001, ***p<0.001, *p<0.05). **iv**, AP amplitude, in mV (no differences among groups). **B**, Ventricular cardiomyocytes. Kir2.1^WT^ (blue; N=3, n=9-15) and Kir2.1^E299V^ (red; N=3, n=11-16). **i**, APD at 20% repolarization for 1, 2, 4, 5 and 10Hz (*p<0.05). **ii**, APD at 50% repolarization for 1, 2, 4, 5 and 10Hz (**p<0.01; *p<0.05). **iii**, APD at 70% repolarization for 1, 2, 4, 5 and 10Hz) (****p<0.0001; ***p<0.001). **iv**, AP amplitude in mV (no significant differences among groups). Mann-Whitney test applied for comparisons.


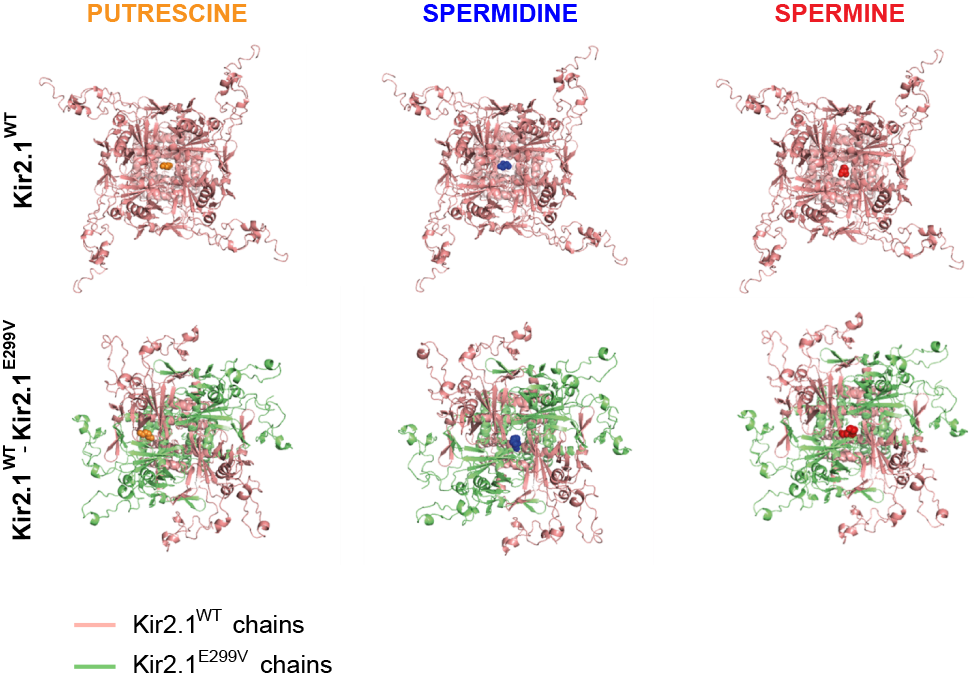


**Supplementary Figure 12. Polyamines fail to block the cytoplasmic pore of Kir2.1^E299V^ channels.** *In-silico* ligand docking of polyamines (putrescine in orange, spermidine in blue and spermine in red) at the cytoplasmic pore of Kir2.1^WT^ channels, and lack thereof for Kir2.1^WT^-Kir2.1^E299V^ channels. Kir2.1^WT^ chains are shown in pink and Kir2.1^E299V^ chains in light green.


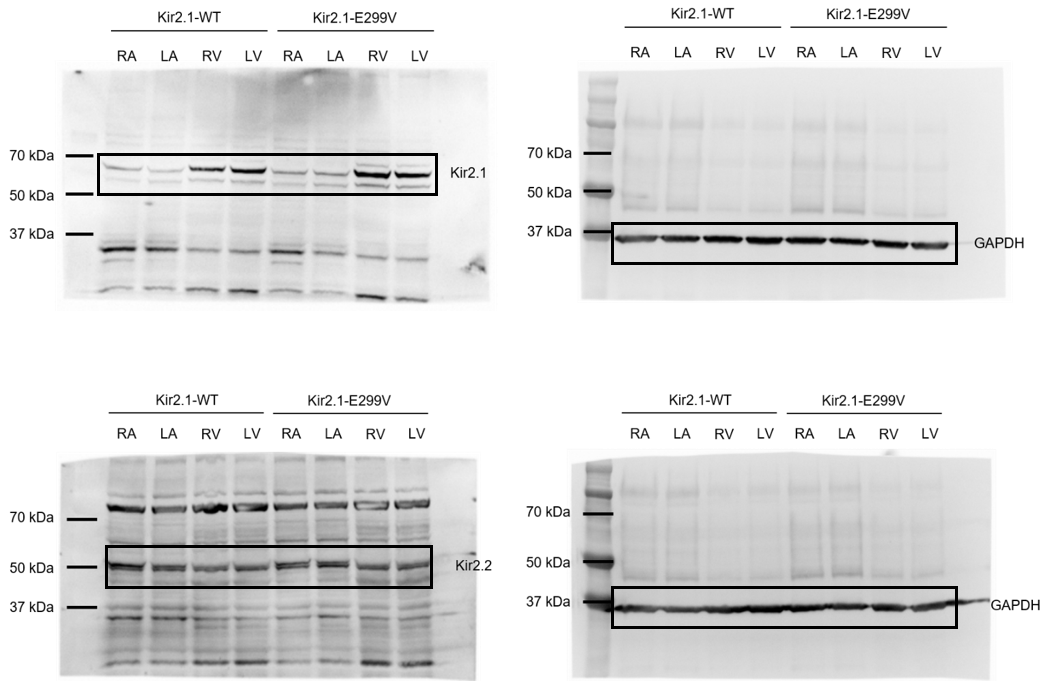


**Supplementary Figure 13.** Uncropped images for western blot gels presented in Figure 6. Black boxes mark the borders of the final cropped images.


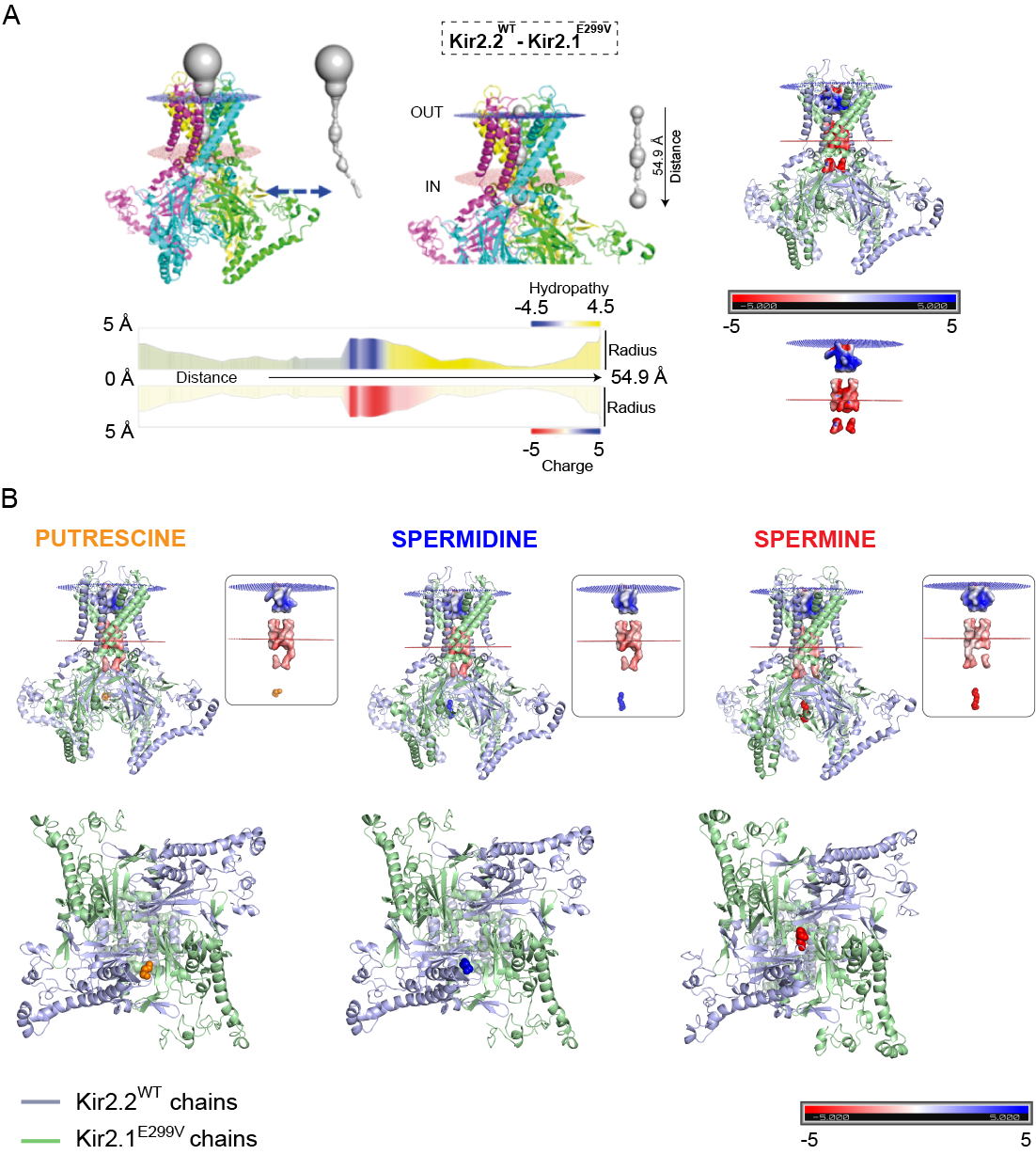


**Supplementary Figure 14. Interaction between Kir2.2^WT^-Kir2.1^E299V^ subunits reveals a reduced channel pore diameter in the atria. A**, Molecular modelling showing the cytoplasmic pore region (blue dashed arrow) of the Kir2.2^WT^-Kir2.1^E299V^ channel. **B**, **Top**, Kir2.2^WT^-Kir2.1^E299V^ interacting with polyamines side view; **bottom**, birds’ eye view from the cytoplasmic side. Polyamines (putrescine in orange, spermidine in blue and spermine in red) fail to penetrate and block the channel. Kir2.2^WT^ chains are in light blue and Kir2.1^E299V^ chains in light green.


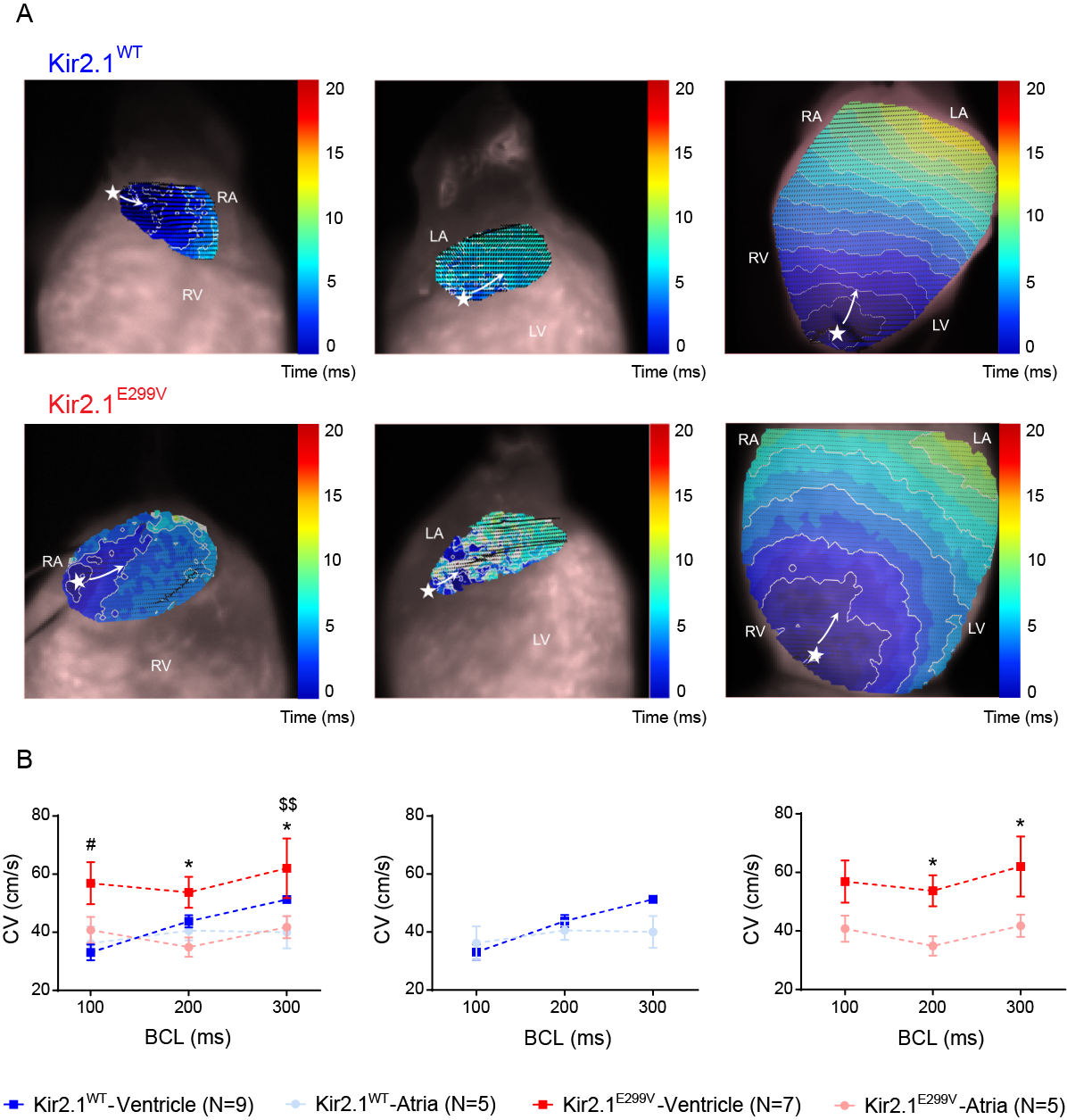


**Supplementary Figure 15. The Kir2.1^E299V^ mutation increases conduction velocity (CV) in the ventricles but not in the atria. A**, Representative 1-ms isochronal activation maps for right atria, left atria and ventricles paced at 300ms basic cycle length (BCL); **top**, Kir2.1^WT^ hearts; **bottom**, Kir2.1^E299V^ hearts. Star, stimulation site. RA and LA indicate right and left atria; RV and LV indicate right and left ventricles. Note that the CV data for the atria is the mean of RA and LA due to the absence of differences between Kir2.1^WT^ and Kir2.1^E299V^. **B**, CV restitution curves for Kir2.1^WT^ and Kir2.1^E299V^ ventricles and atria at 100, 200 and 300ms BCL. **Left**, CVs of Kir2.1^WT^ ventricles (dark blue, N=9), Kir2.1^WT^ atria (light blue, N=5), Kir2.1^E299V^ ventricles (red, N=7), and Kir2.1^E299V^ atria (light red, N=5), showing the significant increase in the CV of the mutant ventricles compared to the other groups. **Middle**, CVs in Kir2.1^WT^ ventricles *vs* atria. **Right**, ventricular *vs* atrial CV in Kir2.1^E299V^ hearts. The larger CV in the Kir2.1^E299V^ ventricles indicates higher excitability than the atria. # statistically significant difference between Kir2.1^WT^ ventricles and Kir2.1^E299V^ ventricles; * statistically significant difference between Kir2.1^E299V^ ventricles and Kir2.1^E299V^ atria; ^$^ statistically significant difference between Kir2.1^E299V^ ventricles and Kir2.1^WT^ atria. Two-way ANOVA was applied.

**
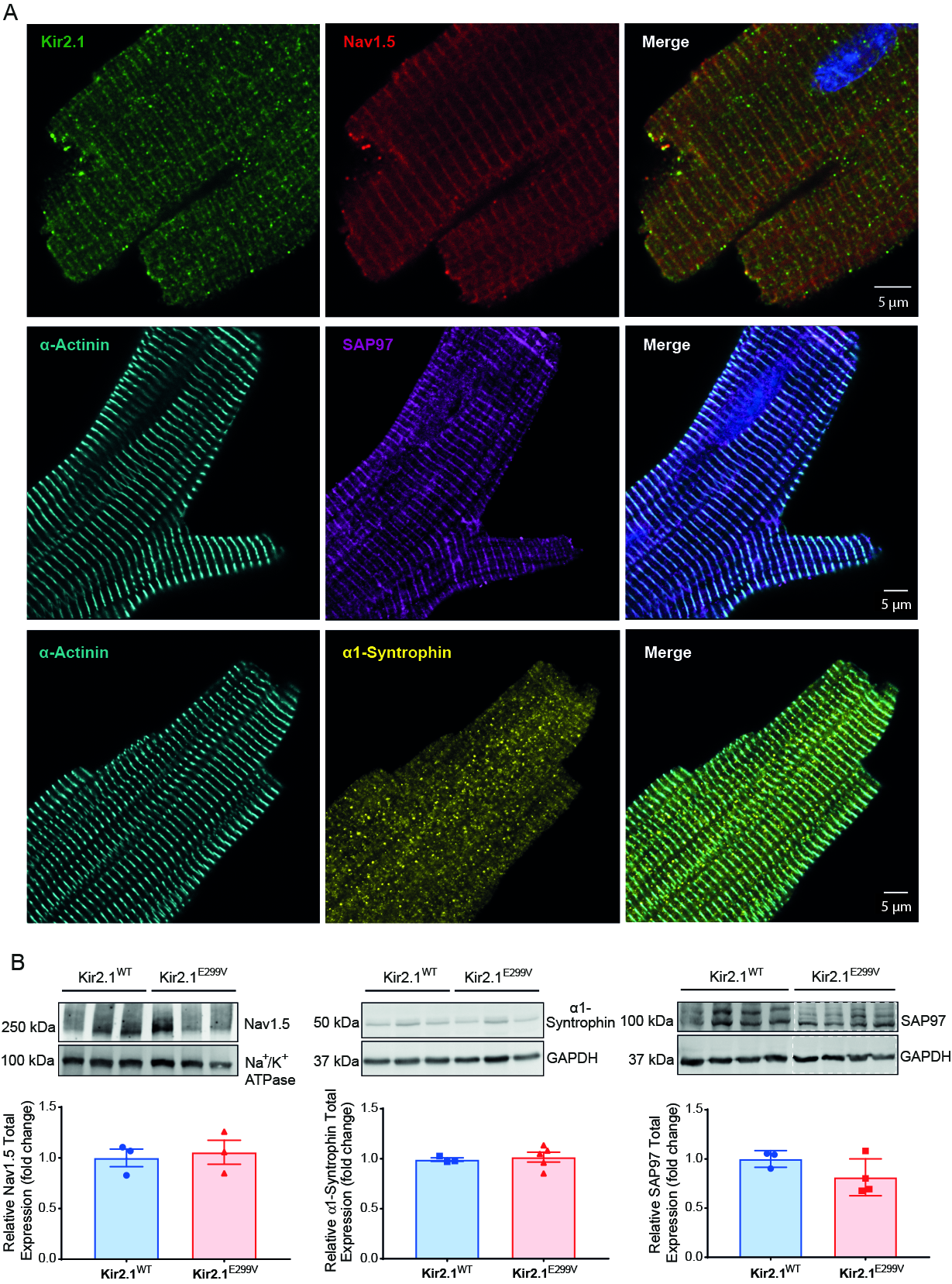
**

**Supplementary Figure 16. Main Kir2.1 interactors are not modified in Kir2.1^E299V^ cardiomyocytes. A**, Kir2.1 (green), Na_v_1.5 (red), SAP97 (purple), α1-Syntrophin (yellow) and α-Actinin (light blue) staining in Kir2.1^E299V^ cardiomyocytes showing the normal distribution pattern of these proteins. Scale bars, 5µm. **B**, Western blot experiments and quantification of Nav1.5, α1-Syndrophin and SAP97 total protein levels in hearts from Kir2.1^E299V^ (red) mice compared to Kir2.1^WT^ (blue). Na^+^/K^+^ ATPase and GAPDH were used as loading controls (N=3-4 animals per condition analysed in triplicate). Mann-Whitney test applied for comparisons.


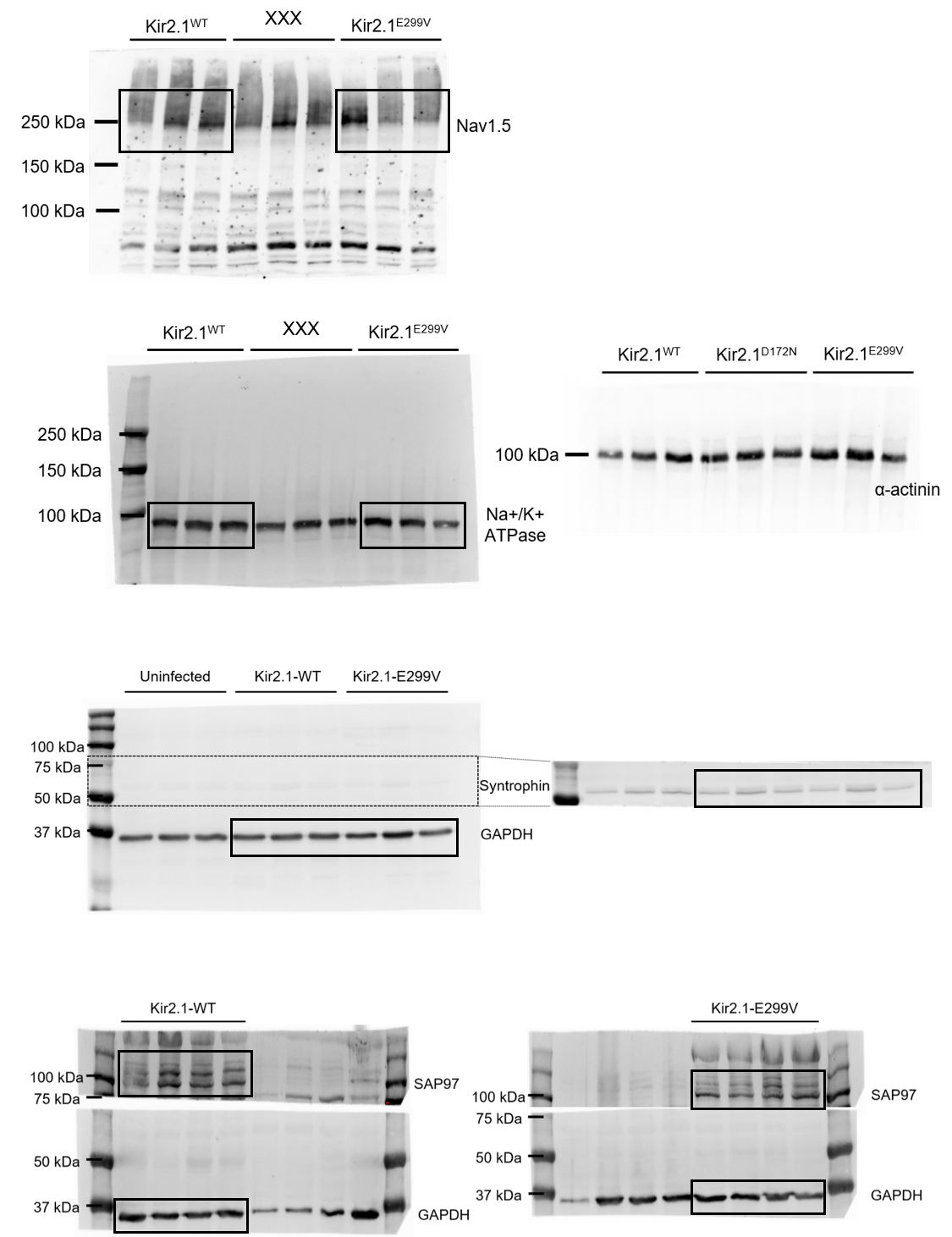


**Supplementary Figure 17.** Uncropped images for western blot gels presented in Supplementary Figure 14. Black boxes mark the borders of the final cropped images. We also present the staining of a second loading control (α-actinin, 100kDa) to confirm the Nav1.5 expression results.

**
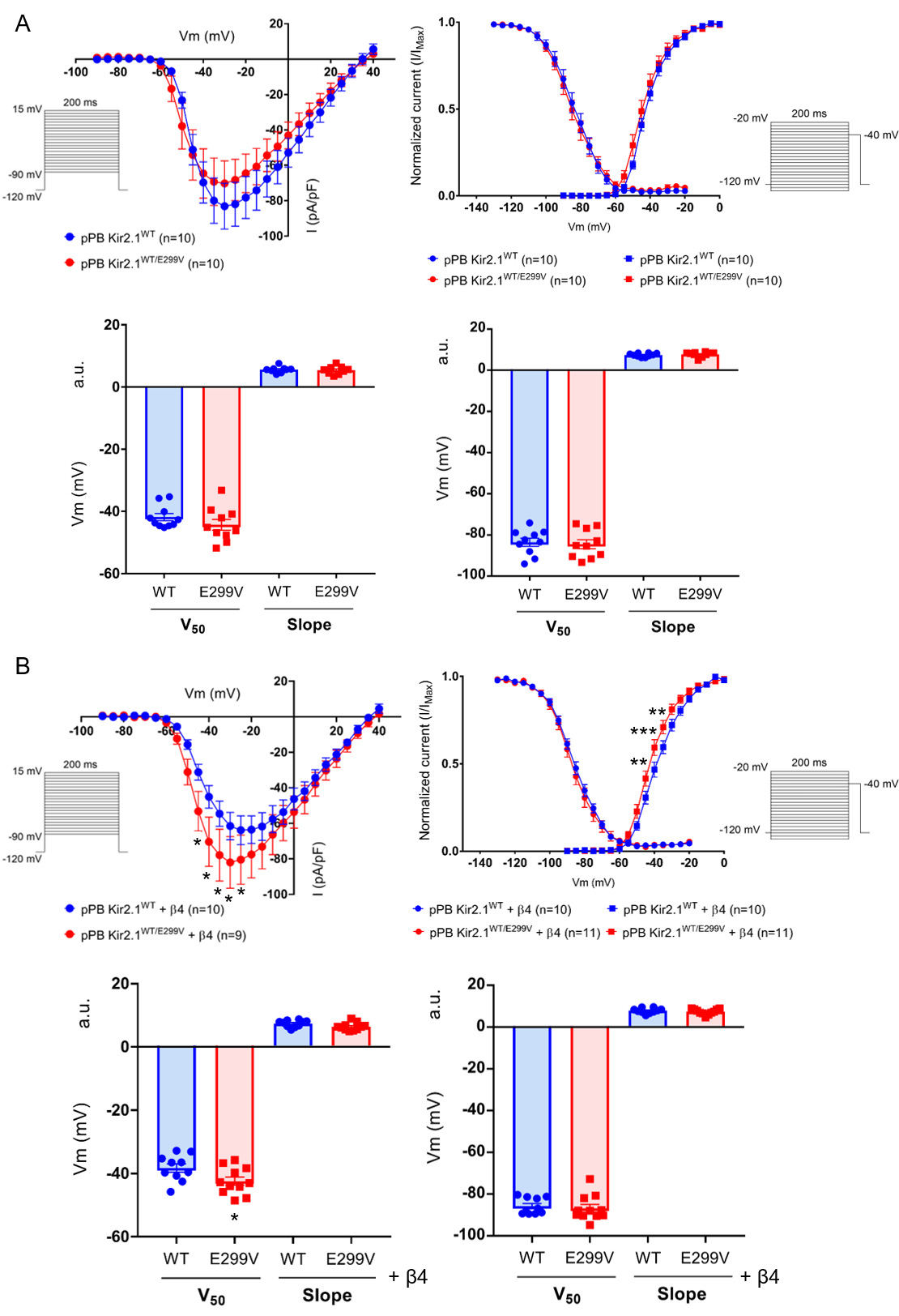
**

**Supplementary Figure 18. Navβ4 subunit modifies I_Na_ density, with a slight shift of the sodium kinetic properties, in heterotetrameric Kir2.1^WT/E299V^ cells.** **A**, HEK-Nav1.5 cells transfected with Kir2.1^WT^ (2µg, in blue) or Kir2.1^WT^ (1µg) / Kir2.1^E299V^ (1µg, in red) simulating the heterozygous condition. Graphs show the I_Na_ density and biophysical kinetics (activation and inactivation curves and parameters). Whole-cell patch-clamp experiments were obtained from 3 independent transfections, with 10 cells per condition. **B**, Current/voltage (IV) relationships from HEK-Nav1.5 cells transfected with Navβ4 (1µg) in addition to Kir2.1^WT^ (2µg, in blue) or Kir2.1^WT^ (1µg) / Kir2.1^E299V^ (1µg, in red) (*p<0.05). Three independent transfections, with 9-11 cells per condition. Two-way ANOVA (IV and activation/inactivation curves) and Mann-Whitney test (activation/inactivation parameters) applied for comparisons.


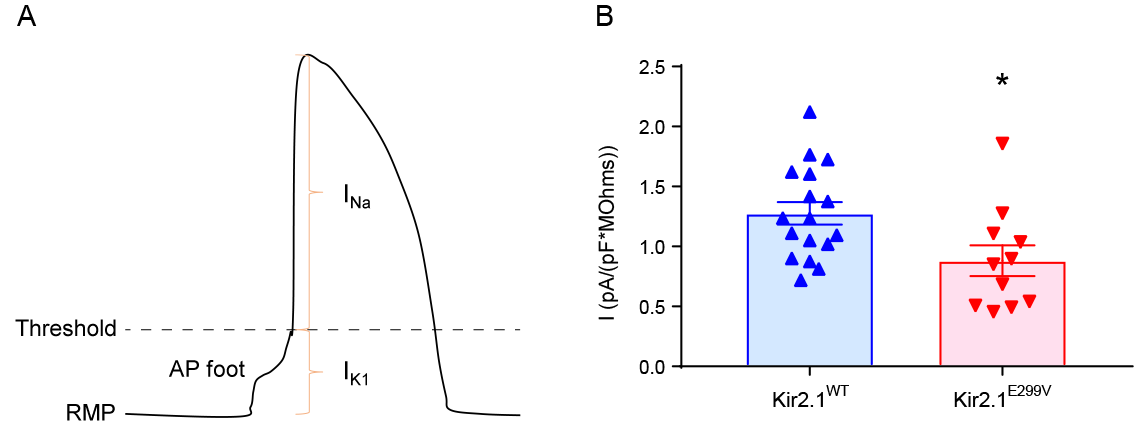


**Supplementary Figure 19. A**, Trace of an stimulated AP with the current needed to reach threshold voltage where we indicate the resting membrane potential (RMP), the foot potential and the threshold, in addition to the currents responsible for its triggering during the phase 0. **B**, Amount of current needed to reach threshold in Kir2.1^WT^ and Kir2.1^E299V^ ventricular cardiomyocytes, normalized by the access resistance**.** 1.28±0.09 pA/(pF*MOhm) for Kir2.1^WT^ (N=3; n=17) *vs* 0.88±0.13 pA/(pF*MOhm) for Kir2.1^E299V^ (N=3; n=11) cardiomyocytes (*p=0.0168). Mann-Whitney test applied for comparisons.

**
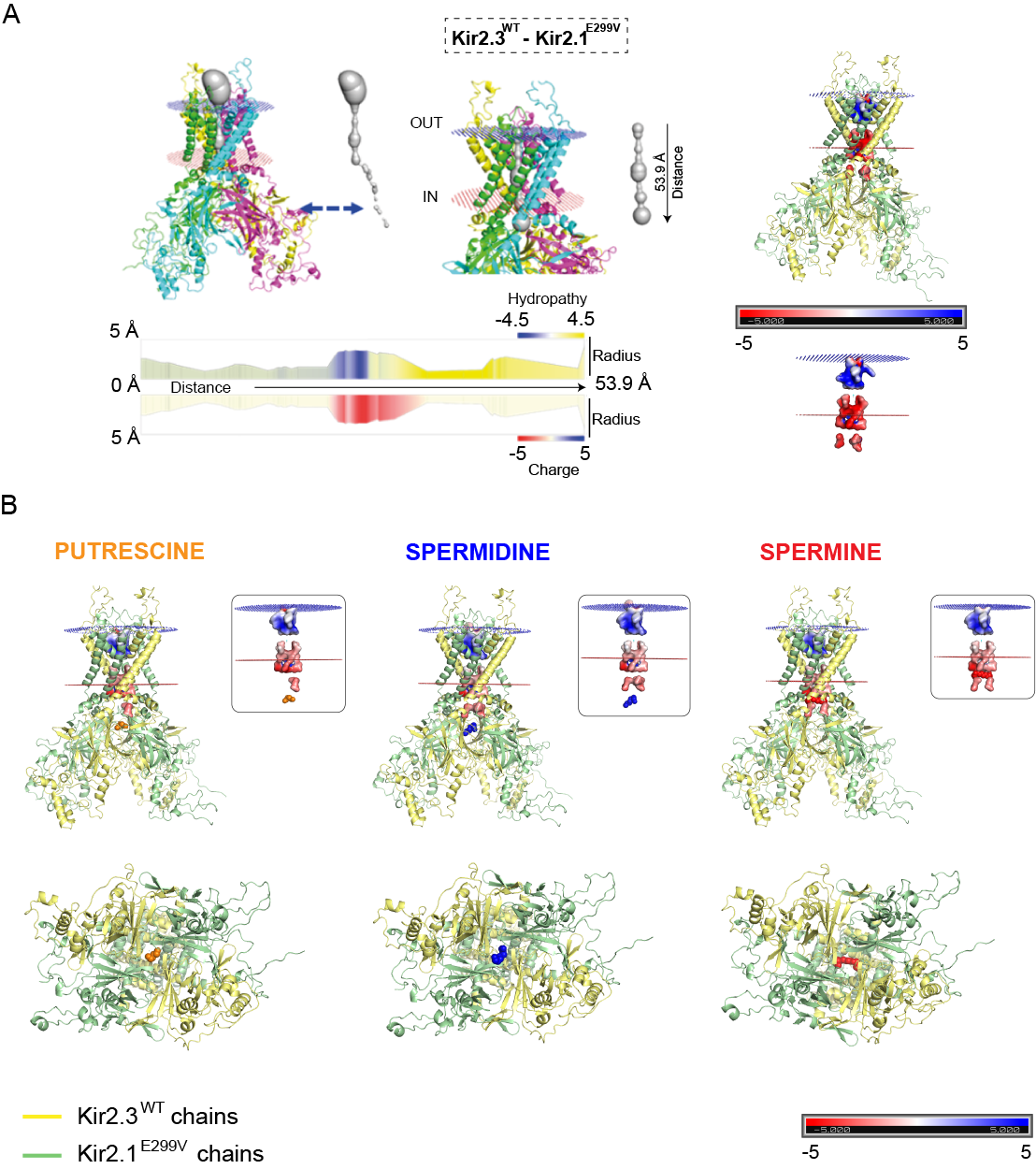
**

**Supplementary Figure 20. Polyamines do not block the cytoplasmic pore of heterotetrameric Kir2.3^WT^-Kir2.1^E299V^ channels. A**, Molecular modelling showing the structural characteristics of the Kir2.3^WT^-Kir2.1^E299V^ pore, in addition to its hydropathy and polarity. **B**, Structural binding of polyamines (putrescine in orange, spermidine in blue and spermine in red) to the channel to analyse their capacity to block the heterotetrameric Kir2.3^WT^-Kir2.1^E299V^ pore (vertical/whole vision in the upper panel and cytoplasmic vision in the lower panel). Kir2.3^WT^ chains are shown in purple and Kir2.1^E299V^ chains in light green.

**SUPPLEMENTARY TABLES**

**Supplementary Table 1. External and internal solutions used in patch-clamp experiments.**

|  | **K^+^ Currents / Action Potentials** | |  |  | | **Na^+^ Currents** | | |  |
| --- | --- | --- | --- | --- | --- | --- | --- | --- | --- |
|  | Bath solution (mM) | Internal solution  (mM) | |  |  | | Bath solution (mM) | Internal solution  (mM) |  |
| **Product** | pH 7.4 (NaOH) | pH 7.2  (KOH) | |  |  | | pH 7.35 (CsOH) | pH 7.2  (CsOH) |  |
| Calcium chloride (CaCl_2_) | 1 | 1 | |  |  | | 1 | - |  |
| Cesium chloride (CsCl) | - | - | |  |  | | 132.5 | - |  |
| Cesium Fluoride (CsF) | - | - | |  |  | | - | 135 |  |
| EGTA | - | 10 / - | |  |  | | - | 10 |  |
| Glucose | 5.5 | - | |  |  | | 10 | - |  |
| HEPES | 10 | 5 | |  |  | | 20 | 5 |  |
| K-Aspartate | - | 110 | |  |  | | - | - |  |
| K_2_ATP | - | 4 | |  |  | | - | - |  |
| Potassium Chloride (KCl) | 5.4 or 30* | 20 | |  |  | | - | - |  |
| Magnesium Adenosine-Tri-Phosphate (MgATP) | - | - | |  |  | | - | 5 |  |
| Magnesium Chloride (MgCl_2_) | 1 | 1 | |  |  | | 1 | - |  |
| Nickel (II) Chloride (NiCl_2_) | - | - | |  |  | | 1 | - |  |
| Sodium Chloride (NaCl) | 130 or 110* | 8 | |  |  | | 5 or 20** | 5 |  |
| Na_2_HPO_4_ | 0.33 | - | |  |  | | - | - |  |

***** Modified bath solution (30mM KCl and 110mM NaCl) for recording I_K1_ from HEK-293T cells transfected with Kir2.1-Kir2.2 dimers.

****** 5mM NaCl in voltage-clamp experiments recorded in murine cardiomyocytes. 20mM NaCl in voltage-clamp experiments performed in transfected HEK-Nav1.5 cells.

**Supplementary Table 2. Primary antibodies used in immunofluorescence experiments.**

| **Target** | **Dilution** | **Company** | **Catalogue number** | **Specie** |
| --- | --- | --- | --- | --- |
| Anti-Kir2.1 | 1:100 | Alomone | APC-026 | Rabbit polyclonal |
| Anti-Nav1.5 | 1:50 | Alomone | AGP-008 | Guinea pig polyclonal |
| Anti-α-Actinin | 1:200 | Sigma | A7732 | Mouse monoclonal |
| Anti-SAP97 | 1:200 | Invitrogen | PA1-741 | Rabbit polyclonal |
| Anti-α1-Syntrophin | 1:200 | Invitrogen | PA5-22357 | Rabbit polyclonal |

**Supplementary Table 3. Secondary antibodies used in immunofluorescence experiments.**

| **Target** | **Dilution** | **Company** | **Catalogue number** | **Specie** |
| --- | --- | --- | --- | --- |
| Anti-Rabbit Alexa Fluor 488 | 1:500 | Invitrogen | A11034 | Goat anti-rabbit IgG |
| Anti-Rabbit Alexa Fluor 568 | 1:500 | Invitrogen | A11036 | Goat anti-rabbit IgG |
| Anti-Mouse Alexa Fluor 488 | 1:500 | Invitrogen | A11029 | Goat anti-mouse IgG |
| Anti-Mouse Alexa Fluor 568 | 1:500 | Invitrogen | A11031 | Goat anti-mouse IgG |
| Anti-Guinea pig DyLight 680 | 1:300 | Invitrogen | SA5-10098 | Goat anti-guinea pig IgG |

**Supplementary Table 4. Primary antibodies used in western blotting experiments.**

| **Target** | **Dilution** | **Company** | **Catalogue number** | **Specie** |
| --- | --- | --- | --- | --- |
| Anti-Kir2.1 | 1:200 | Abcam | Ab109750 | Rabbit monoclonal |
| Anti-Kir2.2 | 1:200 | Alomone | APC-042 | Rabbit polyclonal |
| Anti-Nav1.5 | 1:500 | Sigma | S0819 | Rabbit polyclonal |
| Anti-SAP97 | 1:500 | Invitrogen | PA1-741 | Rabbit polyclonal |
| Anti-α1-Syntrophin | 1:1000 | Invitrogen | PA5-22357 | Rabbit polyclonal |
| Anti-GAPDH | 1:2500 | Abcam | Ab8245 | Mouse monoclonal |
| Anti-Na^+^/K^+^ ATPase | 1:4000 | Abcam | Ab7671 | Mouse monoclonal |
| Anti-α-Actinin | 1:2500 | Sigma | A7732 | Mouse monoclonal |

**Supplementary Table 5. Secondary antibodies used in western blotting experiments.**

| **Target** | **Dilution** | **Company** | **Catalogue number** | **Specie** |
| --- | --- | --- | --- | --- |
| Anti-Rabbit Alexa Fluor 680 | 1:10000 | Invitrogen | A21076 | Goat anti-rabbit IgG |
| Anti-Mouse Alexa Fluor 680 | 1:10000 | Invitrogen | A21057 | Goat anti-mouse IgG |
| Anti-Rabbit HRP | 1:1000 | Invitrogen | 65-6120 | Goat anti-rabbit IgG |
| Anti-Mouse HRP | 1:1000 | Invitrogen | 31430 | Goat anti-mouse IgG |

**Supplementary Table 6. Primers used in RT-qPCR experiments**

| **Protein (Gene)** | **Forward primer (5’-3’)**  **Melting Temperature** | **Reverse primer (5’-3’)**  **Melting Temperature** |
| --- | --- | --- |
| Human Kir2.1-IRES (*KCNJ2*) | GCAAGTGTACCTCTAGAGCCCAG  65.1 °C | CCTTATTCCAAGCGGCTTCG  67.6 °C |
| Murine Kir2.1 (*KCNJ2*) | TTCTCCATCGAGACCCAGAC  64.3 °C | ATCTATTTCGTGAACGATAG  53.4 °C |
| Murine Kir2.2 (*KCNJ12*) | TCCACGGCTTCATGGCAGCC  74.9 °C | GTCCAGTGGGATGTACTCAC  59.4 °C |
| Kir2.3 (*KCNJ4*) | TATGGCATGGGCAAGGAG  64.7 °C | AGCTGCCTCCTCCTCCATC  65.9 °C |
| Acidic ribosomal phosphoprotein P0 (*36b4*) | GCGACCTGGAAGTCCAACTA  64.2 °C | ATCTGCTGCATCTGCTTGG  64.3 °C |

**Supplementary Table 7. Atrial APD_90_ (ms) at different stimulation frequencies.** Values corresponding to the APD_90_ (in ms) for all the stimulation frequencies (in Hz) in atrial cardiomyocytes from Kir2.1^WT^ and Kir2.1^E299V^ mice. We also show the statistical significance after applying Mann-Whitney tests and the sample size for each experiment.

| **Atrial APD_90_ (ms) at different frequencies** | | | | | |
| --- | --- | --- | --- | --- | --- |
|  | **APD_90_ 1 Hz** | **APD_90_ 2 Hz** | **APD_90_ 4 Hz** | **APD_90_ 5 Hz** | **APD_90_ 10 Hz** |
| Kir2.1^WT^ | 50.57±6.31 | 47.62±6.20 | 43.83±7.51 | 42.14±8.17 | 29.35±4.58 |
| Kir2.1^E299V^ | 20.19±1.79 | 18.60±2.07 | 16.61±1.58 | 17.21±2.24 | 16.23±1.94 |
| Significance  Sample size | p<0.0001  N=3 (n=11-14) | p<0.0001  N=3 (n=11-13) | p<0.0001  N=3 (n=10-11) | p=0.0005  N=3 (n=10-11) | p=0.0155  N=3 (n=8-10) |

**Supplementary Table 8. Ventricular APD_90_ (ms) at different stimulation frequencies.** Values corresponding to the APD_90_ (in ms) for all the stimulation frequencies studied (in Hz) in ventricular cardiomyocytes from Kir2.1^WT^ and Kir2.1^E299V^ mice. We also show the statistical significance after applying Mann-Whitney tests and the sample size for each experiment.

| **Ventricular APD_90_ (ms) at different frequencies** | | | | | |
| --- | --- | --- | --- | --- | --- |
|  | **APD_90_ 1 Hz** | **APD_90_ 2 Hz** | **APD_90_ 4 Hz** | **APD_90_ 5 Hz** | **APD_90_ 10 Hz** |
| Kir2.1^WT^ | 99.06±7.45 | 74.41±7.88 | 73.46±7.48 | 63.62±5.93 | 47.44±4.46 |
| Kir2.1^E299V^ | 22.51±2.02 | 20.22±2.34 | 20.10±2.39 | 20.42±2.80 | 20.95±2.76 |
| Significance  Sample size | p<0.0001  N=3 (n=14-16) | p<0.0001  N=3 (n=15-16) | p<0.0001  N=3 (n=14) | p<0.0001  N=3 (n=13-15) | p=0.0002  N=3 (n=9-12) |

**EXTENDED MATERIALS AND METHODS**

**Study Approval.** All experimental and other scientific procedures using animals conformed to EU Directive 2010/63EU and Recommendation 2007/526/EC, enforced in Spanish law under *Real Decreto 53/2013*. Animal protocols were approved by the local ethics committees and the Animal Protection Area of the Comunidad Autónoma de Madrid (PROEX 111.4/20).

**Mice.** 4-week-old C57BL/6J male mice were obtained from Charles River Laboratories. Mice were reared and housed in accordance with institutional guidelines and regulations. The mice had access to food and water *ad libitum*. Transgenic Cx40^GFP^ mice ^1-5^ were used for experiments in which we analyzed cardiac conduction system cells at the electrophysiological level. 15-25 weeks-old mice were used for experiments. Experimental procedures were conducted while the animal was under anesthesia, which is known to pause the circadian clock ^6^. Nevertheless, experiments in which the circadian rhythm could be relevant (ECGs, echocardiography and intracardiac stimulation) were done during daylight hours (specifically, from 9 am to 2 pm). On the other side, in some experiments (patch-clamping), the same investigator performed all procedures, including animal handling, data analysis and presentation. Nonetheless, for comparisons between groups, data were extracted automatically from the software used, so no subjective judgment was involved at any step.

**Adeno-associated virus (AAV) production and purification.** Briefly, this was achieved by first generating enhanced tdTomato-reporter AAV vectors driven from the cardiomyocyte-specific cardiac TroponinT proximal promoter (cTnT), strengthened by a *cis*-regulatory motif number 4 enhancer (CRM4) ^7^, and encoding wildtype Kir2.1 (Kir2.1^WT^), or the SQTS3 Kir2.1 mutant (Kir2.1^E299V^). These AAV plasmids were packaged into AAV serotype 9 (AVV9) capsids with the use of pAdDF6 helper plasmids (providing the three *adenoviral helper* genes) and pAAV2/9 (providing *rep* and *cap* viral genes), obtained from PennVector ^8^ by the CNIC Viral Vectors Unit. AAV vectors were produced by the triple transfection method, using HEK293A cells as described previously ^9, 10^. AAV vector titer (viral genomes (vg) per ml) was carried out by quantitative real-time PCR as described ^11^. Known copy numbers (10^5^–10^8^) of the respective plasmid (pAAV-empty vector, pAAV-Kir2.1^WT^ and pAAV-Kir2.1^E299V^) were used to construct standard curves. We administered viral particles (3.5x10^10^ per animal) to 4- to 5-week-old mice i.v. via the femoral vein.

**AAV injection.** Mice were anesthetized with Ketamine (60mg/kg) and Xylazine (20mg/kg) administered i.p. Once asleep, animals were placed on a heated pad at 37±0.5ºC to prevent hypothermia. Thereafter, 3.5x10^10^ vg were inoculated through the femoral vein in a final volume of 50μL, taking care to prevent the introduction of air bubbles. Animals were then maintained on the heating pad until recovery.

**AAV-Mediated Gene Distribution.** Corporal distribution of protein expression was assessed as described ^8^. Briefly, 8 weeks after the AAV9 administration, the *ex vivo* fluorescent signal was determined in infected mice confirming cardiac expression of protein tdTomato used as the reporter gene in the viral construct. The infection rate in mouse hearts (vg per cell) was quantified blindly by immunohistochemistry using an anti-tdTomato antibody. For the analysis, we used a custom-made plugin for Fiji/UmageJ, as previously described ^8^.

**Echocardiography**. Mice were lightly anesthetized with 0.5-2% isoflurane in oxygen, and placed on a 37ºC heating platform in the supine position. A base-to-apex ECG was continuously used to monitor the animal, adjusting isoflurane delivery to maintain the heart rate at 450±50 beats per minute (bpm). Warmed ultrasound gel was used to maintain normothermia. Transthoracic echocardiography was performed blindly by an expert operator from the CNIC Advance Imaging Unit using a high-frequency ultrasound system (Vevo 2100, Visualsonics Inc., Canada) with a 40-MHz linear probe. Two-dimensional (2D) guided M-mode echocardiograms were obtained at a frame rate above 230 frames/sec, and pulse wave Doppler (PW) was acquired at a frequency of 40 kHz. Images were transferred to a computer and analyzed blindly offline using the Vevo 2100 Workstation software. The M-mode sample gate was placed at the level of the papillary muscles. For left ventricle (LV) systolic function assessment, parasternal standard long and short axis views (PSLAX and PSAX view, respectively) were acquired. LV ejection fraction (EF) and chamber dimensions were calculated from these views in systole and diastole.

**Surface electrocardiographic (ECG) recordings.** Mice were anesthetized adjusting isoflurane (0.8­1% volume in oxygen) delivery to maintain the heart rate at 350±50bpm and the efficacy of the anesthesia was monitored by watching breathing speed. A subcutaneous 23-gauge needle electrode connected to an MP36R amplifier (BIOPAC Systems) was attached to each limb, and six-lead surface ECGs were recorded for 5 minutes. We tested the response to 5mg/kg isoproterenol 1.5 minutes after its administration due to its direct and rapid effect on the ECG (Isoprenaline hydrochloride, Sigma), 20mg/kg flecainide during 6 minutes of recordings (10mg/ml Apocard, injectable solution, Mylan) and 40mg/kg chloroquine during 40 minutes of recordings (Chloroquine diphosphate salt, Sigma), all administered intraperitoneally (i.p.). We used AcqKnowledge 4.1 software to analyze, blindly and in pairs, the ECG waves, segments and intervals durations. We applied Bazett’s and Framingham’s formulas to correct the QT interval considering the heart rate.

***In-vivo* intracardial electrophysiological studies***.* After anesthesia (Ketamine 60mg/kg and Xylazine 20mg/kg i.p.), an octopolar catheter (Science) was inserted through the jugular vein and advanced into the right atrium (RA) and right ventricle (RV), as described previously ^12, 13^. His bundle was identified as a small spike between the atrial and ventricular deflections in the intracardiac electrogram (Supplementary Figure 6), as described previously ^14^. For studying the refractory period, we used an S1-S2 protocol consisting of a train of 10 stimuli (S1) at a cycle length of 100ms followed by an extra-stimulus (S2) reducing the S1-S2 interval in 2-ms steps starting at 100ms. Atrial and ventricular arrhythmia inducibility was assessed by applying 10 stimuli at 10Hz (S1), followed by 10 stimuli 20Hz (S2). AF was defined as the occurrence of rapid and fragmented atrial electrograms (lack of P waves) with irregular atrial-ventricular (AV)-nodal conduction and ventricular rhythm. Ventricular tachycardia/ventricular fibrillation (VT/VF) was defined as an excessively fast monomorphic/polymorphic ventricular rhythm independent of atrial excitation.

**Optical mapping in isolated hearts.** We used hearts from Kir2.1^WT^ and Kir2.1^E299V^ mice. Without knowing the genotype, after cervical dislocation, the hearts were rapidly excised through thoracotomy and subsequently connected to a Langendorff perfusion system to be continuously perfused with warm oxygenated Tyrode’s solution (pH 7.4), bubbled with 95% O_2_:5% CO_2_. Hearts were placed in a custom-made plastic chamber maintained at 36 ± 1°C and allowed to equilibrate for 10 minutes. The potentiometric dye Di-4-ANEPPS (Molecular Probes) was added to the perfusated as a bolus to achieve a final concentration of 10μmol/L. We used an optical mapping system comprised of a custom-made upright 128 × 128 pixel eVolve EMCCD camera (Photometrics) running at 1,000 frames per second. Blebbistatin 10μmol/L was used to reduce contraction. Phase, conduction velocity and activation maps were analyzed blindly using custom-made MATLAB software. Right ventricle and atria stimulation was conducted using a custom-made bipolar electrode (≈700μm interelectrode distance) connected to a programmed stimulator (Cibertec). Usually, 10 pulses (amplitude, 8V; duration, 2ms) at 5Hz followed by 10 similar pulses at 25Hz were applied.

**Cardiomyocyte isolation.** To isolate atrial and ventricular cardiomyocytes we used the procedure of Macías *et al* ^15^. Briefly, after cervical dislocation, the mouse heart was isolated, mounted on a modified Langendorff-perfusion apparatus and the aorta was retrogradely perfused with Ca^2+^-free perfusion buffer (PB) containing (in mmol/L): 113 NaCl, 4.7 KCl, 0.6 KH_2_PO_4_, 0.6 Na_2_HPO_4_, 1.2 MgSO_4_·7H_2_O, 12 NaHCO_3_, 10 KHCO_3_, 0.032 Phenol Red, 9.8 HEPES, 30 taurine, 5.5 glucose, 10 2,3-butanedione-monoxime (pH 7.4 with NaOH). Perfusion rate was 1mL/min for 7 min at 37°C. Enzymatic digestion was then started for 10 minutes for the atria and 20 minutes for the ventricles with digestion buffer (DB) consisting of PB supplemented with Liberase™ (0.2mg/mL), Trypsin 2.5% (5.5mmol/L), and CaCl_2_ (12.5µM) at 37°C. Thereafter, atrial or ventricular cardiomyocytes were gently disaggregated and isolated in 3mL DB. The resulting cell suspension was filtered through a 200µm sterile mesh (SEFAR-Nitex) and transferred for enzymatic inactivation to a tube containing 12ml stopping-buffer-1 (SB-1), PB supplemented with fetal bovine serum (FBS, 10% v/v) and CaCl_2_ (12.5µmol/L). After gravity sedimentation for 20 minutes, the supernatant was removed and cardiomyocytes were resuspended in stopping-buffer-2 (SB-2) containing the same concentration of CaCl_2_, but lower FBS (5% v/v) for another 20 minutes. Cardiomyocyte Ca^2+^-reintroduction was performed in SB-2 with two progressively increased CaCl_2_ concentrations (0.112 and 1mmol/L). Cells were resuspended and allowed to decant for 15 min in each step, contributing to the purification of the cardiomyocyte suspension.

**HEK-293T/HEK-Nav1.5 cells culture and transfection**. We maintained HEK-293T cells (ATCC number CRL-3216) in DMEM medium supplemented with 10% FBS, 1% Penicillin/Streptomycin and 1% L-glutamine. We transfected these cells using JetPRIME transfection reagent (Polyplus) with the following plasmids: ITR-cTnT/CMV-*KCNJ2*^WT or E299V^-IRES-*Tomato/GFP*-pA-ITR (ITR means Inverted Terminal Repeats; cTnT refers to the Cardiac Troponin T promoter; CMV refers to Cytomegalovirus promoter; IRES means Internal Ribosome Entry Site; GFP is the Green Fluorescence Protein; and pA refers to poly-Adenosine tail). Non-viral piggy-bac vector (2µg) encoding heterodimer Kir2.1^WT^-Kir2.2 or Kir2.1^E299V^-Kir2.2 were transfected in HEK-293T cells for 48 hours according to the JetPRIME instructions. After 48 hours from the transfection, we carried out the corresponding experiments. We used the same maintenance procedure for HEK293T-Nav1.5 cell line (kindly provided by Dr. Carmen Valenzuela from Dr. Hugues Abriel’s laboratory) but adding 0.2% (200µg/ml) Zeocin in order to select correctly Nav1.5 containing cells ^16^.

**Patch-clamping of isolated cardiomyocytes and HEK cells**. Whole-cell and inside-out patch-clamp techniques, as well as data analysis procedures were similar to those described previously ^17-21^. The external and internal solutions are listed in Supplementary Table 1 ^20, 22^.

Cardiomyocytes were placed in a superfusion chamber (RC-26, Warner Instruments) mounted on the stage of an inverted microscope (DMi8, Leica). Cells were allowed to settle on the bottom of the superfusion chamber before being superfused with the corresponding solution. After patch rupture, whole-cell voltage or current-clamp recordings were made using an Axopatch 200B Amplifier (Axon Instruments) and a MultiClamp 700B Microelectrode Amplifier (Molecular Devices). Pipettes made from borosilicate glass (GD-1, Narishige, OD: 1mm; ID: 0.6mm) had resistances of 2-5MΩ after using the internal solutions specified in Supplementary Table 1. Series resistance compensation of 80-90% was achieved. All voltage-clamp currents were low-pass filtered at 2KHz with an analogue filter and digitized at 4-10Hz. Data were recorded using pClamp 10.6 software with the Clampex 10.6 program and analyzed by the Clampfit 10.6 program (Axon Instruments, Foster City, USA). Current amplitudes were normalized to the cell capacitance to account for differences in cell size and expressed as densities (pA/pF).

Action potential (AP) recordings: Threshold current was determined using 1ms pulses at increasing amplitudes (0.2nA/pulse) and frequency of 1Hz. Thereafter, AP were evoked by the injection of 1ms pulses of constant amplitude. AP duration (APD) was measured at 20, 50, 70 and 90% of repolarization.

Current-voltage (IV) relationships:

- *Potassium currents*. IV relationship for the inwardly rectifying K^+^ current (I_K1_) densities were constructed from the current changes produced by a 500-ms voltage-clamp step applied in 10mV increment from -110 to +50 mV from a holding potential of -80mV at 0.1Hz at room temperature (RT). I_K1_ was calculated by subtracting currents recorded in the absence or presence of 500µM BaCl_2_ and relativized to their respective capacitance. For experiments with HEK cells, we modified lightly the external solution in order to promote the I_K1_ current: 30mM KCl and 110mM NaCl instead of 5.4mM and 130mM, respectively. In this way, the reversal potential of this experiments shifts towards more positive potentials (from -80 to -30mV).
- *Sodium currents*. To record the sodium current (I_Na_) density-voltage relationship, cells were held at -120mV and stepped for 100ms from -90mV up to +15mV in 5mV increments at 0.2Hz. I_Na_ was measured at the peak. To analyze the inactivation, cells were held at -120mV and stepped for 100ms from -140mV up to -20mV in 10mV increments at 0.2Hz. In both cases, leak currents were subtracted using the P/4 protocol. Steady-state activation and inactivation curves were fitted by using a Boltzmann equation, in which V_50_ is half-maximum (in)activation potential and k is the Slope factor.

Inside-out patch clamp: Ventricular cardiomyocytes settled on the bottom of the perfusion chamber were superfused with a solution containing (in mM): 123 KCl, 5 EDTA, 7.2 K_2_HPO_4_, and 8 KH_2_PO_4_ (pH 7.2 KOH). The pipette solution contained a low potassium concentration: (in mM) 5 EDTA, 7.2 K_2_HPO_4_ and 8 KH_2_PO_4_. Briefly, after establishing the cell-attach configuration with a high-resistance seal, we lifted the cell to obtain a patch of membrane into the inside-out configuration. In those cases, in which the inside-out patch was not directly got, a brief exposure to air was applied. We then recorded basal protocol (holding at 0mV and stepped for 500ms from +60mV to -120mV in 10mV decrements) and voltage protocol in a ramp (holding at 0mV and ramp from -140mV to +140mV in 1s), and did the same after adding 1mM MgCl_2_, 25ug/ml PIP_2_ and modifying the spermine concentration (10µM, 30µM, 100µM, 300µM and 1mM).

**Immunohistochemical procedure.** We analyzed the amount of AAV9 infection in mouse hearts (see above). After washing with PBS, the heart was fixed in 4% paraformaldehyde (PFA) in PBS and conserved in 70% ethanol. Samples were mounted in Optimal Cutting Temperature (OCT) embedding compound and frozen at -20 to -80°C. We cut 5-7µm thick tissue sections using a cryostat and thaw-mounted the sections onto gelatin-coated histological slides. After permeating (0.2% Triton X-100 in PBS) and blocking (10% normal goat serum in PBS), we used the goat polyclonal anti-tdTomato antibody (Sicgen, AB8181-200) overnight and the anti-goat HRP secondary antibody. We also incubated the samples with Hematoxylin-Eosin to study possible structural changes in infected compared with uninfected hearts.

**Immunofluorescence.** Isolated cardiomyocytes were washed using PBS and fixed with 4% PFA in PBS. Cardiomyocytes were permeabilized using Triton X-100 0.2% in PBS and then blocked in suspension with 10% normal goat serum (NGS) in a solution containing Triton X-100 0.1% in PBS. We incubated the cells with primary antibodies overnight 4ºC and secondary antibodies (1/500 in all cases) during one hour at RT (the antibodies and their dilutions are specified in Supplementary Tables 2 and 3). We washed three times the samples after each antibodies’ incubations with PBS. Samples were mounted in Fluoroshield™-DAPI imaging medium (F6057, Merck). Images of individual cardiomyocytes were acquired with a Leica SP8 confocal microscope.

**Western blot and membrane fractionation procedures.** To analyze total expression levels, we excised atria and ventricles from uninfected, Kir2.1^WT^ and Kir2.1^E299V^ and lysed them in ice-cold RIPA buffer (150mM NaCl, 10mM Tris-HCl, 1mM EDTA, 1% Triton X-100, 0.1% SDS and 0.1% Sodium deoxycholate). We sonicated the samples for 15s and conserved them in ice for 30 minutes, applying 10s of vigorous shaking every 10 minutes. We centrifuged the samples for 20 minutes at 21000g and then recovered the supernatant. We quantified the resulting samples using Pierce™ BCA Protein Assay Kit (Thermo Fisher) and 25-80µg of protein resolved in 5-10% SDS-PAGE gels. We carried out the transference with the Trans-Blot Turbo Transfer System (Bio-Rad, 1704150). The antibodies we employed are listed in tables 3 and 4 (Supplementary Tables 4 and 5).

To determine the Kir2.1 protein levels at the plasma membrane, we used isolated mouse cardiomyocytes and followed the manufacturer’s instructions for the Plasma Membrane Protein Extraction Kit (Abcam, ab65400). Briefly, we separated the cytoplasmic fraction and, after different centrifugations, we obtained the fraction corresponding to plasma membrane proteins. We resolved total, cytosolic and plasma membrane fractions pertaining to 3 animals per condition in 10% SDS-PAGE gels. We used a rabbit monoclonal anti-Kir2.1 antibody 1:200 (Abcam, ab109750). As a protocol limitation, we have slight contamination between fractions and, consequently, a residual expression of membrane proteins in the cytosolic fraction, and *vice versa*.

**Quantitative real-time PCR (qRT-PCR).** Heart samples from uninfected, AAV9-Tomato, AAV9-Kir2.1^WT^ and AAV9-Kir2.1^E299V^ mice were homogenized with the Trizol procedure for RNA extraction (ThermoFisher, 15596026). RNA concentration was measured using UV spectrophotometry at 260nm (Nanodrop 2000, Thermo Scientific). cDNA was obtained using the High Capacity cDNA Reverse transcription Kit (Applied Biosystems, 4368814), avoiding DNA contamination with DNase treatment of RNA samples prior to qRT-PCR. qPCR was performed using SYBR Green PCR Master Mix (Takara, RR420). Primer sequences used were listed in Supplemental Table 6. Each reaction was run in triplicates. Gene expression values were normalized to the average expression of housekeeping gene 36b4.

**Computational modeling**. Fasta sequences of distinct mature Kir2.x isoforms and Kir2.1^E299V^ mutant where aligned to Uniclust·30 (release 08-2018) and pdb70 (release 09-2019) databases using the *hhblits/hhsearch* tools of the HH-suite3^23^ to obtain a multi-sequence alignment (MSA) and pdb templates for comparative modeling.

To dock the full tetramer for each case, a comparative modeling was made using de *RosettaCM* ^24^ tool of the Rosetta suite v3.11 (www.rosettacommons.org) with the fasta sequence, the alignment and templates obtained before for each one within a run of ~1000 decoys to output the desire structures.

In each case, the model with best structural alignment to the templates and minimal energy was selected as final candidate. A final cycle of refinement to minimize clashes and energy was made with the *mp relax* tool using the *rosetta script* interface ^25, 26^ of Rosetta suite v3.11 (www.rosettacommons.org). This tool recomputes the side-chain coordinates of the protein residues accounting for the membrane environment, the lipophilicity, the trans-membrane (TM) segment and the composition of the protein. At least, 100 independent models were calculated for each one. As before, the models with best energy and correct folding were selected as final models. The model with correct topology and minimal score was selected as the final model. Membrane proteins were analyzed according to their interface interactions. Measures were made in Rosetta Energy units (REU), where 1 REU = 1 kcal/mol.

To better show the protein position and orientation in the membrane, the models were submitted to the PPM server ^27^ (http://opm.phar.umich.edu/). This server is specialized in predicting and positioning membrane proteins from 3D structures using a large structural database (membranome) and computational methods. The method accounts for long-range electrostatic interactions, first-shell solvation energy (van der Waals, hydrophobic and hydrogen bond interactions), the gradual polarity changes along the bilayer normal, the preferential solvation of protein groups by water, and the hydrophobic mismatch for TM proteins. Pore evaluation is made with *MOLE* toolkit ^28^ that allows the study of the TM channel and full channel in biomacromolecular structures. Analysis of Poisson-Boltzmann electrostatics calculations are made with *PDB2PQR - APBS* tool suite ^29, 30^. The evaluation of the electro-potential and electrostatic forces considered the lining residues from the central axe of the channel.

**Ligand-docking**. To compare the effect of different ligands (putrescine, spermidine and spermine), the complex of each one with the channel was modelled before they were docked with the *ligand-docking* tool using the *rosetta script* of the Rosetta software suite v3.11. Briefly, a representative conformer of the ligand was located close to the channel in the receptor. The protocol computes combinations of atomic coordinates for conformers of ligand and side-chain rearrangements (rotamers) of the residues to explore the conformational space and find interactions between ligand and protein. As before, for each model, the electro-potential of the environment around both TM and full channels, and the electro-potential for the lining residues from the central axis of the channel were calculated using the *APBS v1.5* ^29^ and *PDB2PQR v2.1.1* ^30^ tools. In all cases, at least 1000 models were computed and the model with correct interface topology (ligand inside the pocket without clashes between ligand and protein) and best interface energy (minor ΔG, more stability and interactions between protein and ligand like electrostatic, hydrophobic/hydrophilic, van der Waals and hydrogen bonds or salt bridges) was selected. A final cycle of refinement to minimize clashes and energy was made with the *mp relax* tool using the *rosetta script* interface ^25, 26, 31^ of Rosetta suite v3.11 (www.rosettacommons.org). The model with the correct topology and minimal score was selected as the final model and was submitted to the PPM server ^27^ (http://opm.phar.umich.edu/). To evaluate the effect of the mutation in homo- and heterotetramers on the channel, TM or full channel were calculated for each model using the *Mole 2.5* tool ^28^.

**Statistical analysis**. We used GraphPad Prism software version 7.0 and 8.0. We applied the statistical tests after a normal (Gaussian) distribution analysis (Shapiro-Wilk normality test and equality of variances with F test). With 2 groups and normal distribution, comparisons were made using unpaired 2-tailed Student’s t-test. When the data do not follow a Gaussian distribution, we apply the nonparametric Mann-Whitney test. Unless otherwise stated, we used one- or two-way ANOVA followed by the post hoc Tukey test for comparison among more than two groups. If data did not follow a normal distribution, the nonparametric equivalent Kruskal-Wallis test was performed. To exclude outliers from some data sets we performed Grubbs' test, also known as the ESD (extreme studentized deviate) method, to determine whether a given value was a statistically significant outlier from the rest. For ECG recordings, we used 10-20 mice per condition and the analysis were carried out by two blinded researchers; on intracardiac stimulation procedures, around 10 animals of each genotype were used; and about 10-15 cells from 3 mice or independent transfections (when working with HEK cells) were studied in patch-clamp experiments. Unless other was specified, “N” means number of mice (or transfection) and “n” means the samples or cells analyzed in each case. Data are expressed as mean ± SEM, and differences are considered significant at p<0.05 (*p<0.05; **p<0.01; ***p<0.001; ****p<0.0001).

Graphical abstract was created using Biorender (https://www.biorender.com/).

**REFERENCES**

1. Anumonwo JM, Tallini YN, Vetter FJ, Jalife J. Action potential characteristics and arrhythmogenic properties of the cardiac conduction system of the murine heart. *Circ Res* 2001;**89**:329-335.

2. Cerrone M, Noujaim SF, Tolkacheva EG, Talkachou A, O'Connell R, Berenfeld O, Anumonwo J, Pandit SV, Vikstrom K, Napolitano C, Priori SG, Jalife J. Arrhythmogenic mechanisms in a mouse model of catecholaminergic polymorphic ventricular tachycardia. *Circ Res* 2007;**101**:1039-1048.

3. Herron TJ, Milstein ML, Anumonwo J, Priori SG, Jalife J. Purkinje cell calcium dysregulation is the cellular mechanism that underlies catecholaminergic polymorphic ventricular tachycardia. *Heart Rhythm* 2010;**7**:1122-1128.

4. Kang G, Giovannone SF, Liu N, Liu FY, Zhang J, Priori SG, Fishman GI. Purkinje cells from RyR2 mutant mice are highly arrhythmogenic but responsive to targeted therapy. *Circ Res* 2010;**107**:512-519.

5. Vaidyanathan R, O'Connell RP, Deo M, Milstein ML, Furspan P, Herron TJ, Pandit SV, Musa H, Berenfeld O, Jalife J, Anumonwo JM. The ionic bases of the action potential in isolated mouse cardiac Purkinje cell. *Heart Rhythm* 2013;**10**:80-87.

6. Poulsen RC, Warman GR, Sleigh J, Ludin NM, Cheeseman JF. How does general anaesthesia affect the circadian clock? *Sleep Med Rev* 2018;**37**:35-44.

7. Utrilla RG, Nieto-Marin P, Alfayate S, Tinaquero D, Matamoros M, Perez-Hernandez M, Sacristan S, Ondo L, de Andres R, Diez-Guerra FJ, Tamargo J, Delpon E, Caballero R. Kir2.1-Nav1.5 Channel Complexes Are Differently Regulated than Kir2.1 and Nav1.5 Channels Alone. *Front Physiol* 2017;**8**:903.

8. Cruz FM, Sanz-Rosa D, Roche-Molina M, Garcia-Prieto J, Garcia-Ruiz JM, Pizarro G, Jimenez-Borreguero LJ, Torres M, Bernad A, Ruiz-Cabello J, Fuster V, Ibanez B, Bernal JA. Exercise triggers ARVC phenotype in mice expressing a disease-causing mutated version of human plakophilin-2. *J Am Coll Cardiol* 2015;**65**:1438-1450.

9. Xiao X, Li J, Samulski RJ. Production of high-titer recombinant adeno-associated virus vectors in the absence of helper adenovirus. *J Virol* 1998;**72**:2224-2232.

10. Hauswirth WW, Lewin AS, Zolotukhin S, Muzyczka N. Production and purification of recombinant adeno-associated virus. *Methods Enzymol* 2000;**316**:743-761.

11. Prasad KR, Xu Y, Yang Z, Toufektsian MC, Berr SS, French BA. Topoisomerase Inhibition Accelerates Gene Expression after Adeno-associated Virus-mediated Gene Transfer to the Mammalian Heart. *Mol Ther* 2007;**15**:764-771.

12. Bao Y, Willis BC, Frasier CR, Lopez-Santiago LF, Lin X, Ramos-Mondragon R, Auerbach DS, Chen C, Wang Z, Anumonwo J, Valdivia HH, Delmar M, Jalife J, Isom LL. Scn2b Deletion in Mice Results in Ventricular and Atrial Arrhythmias. *Circ Arrhythm Electrophysiol* 2016;**9**.

13. Clasen L, Eickholt C, Angendohr S, Jungen C, Shin DI, Donner B, Furnkranz A, Kelm M, Klocker N, Meyer C, Makimoto H. A modified approach for programmed electrical stimulation in mice: Inducibility of ventricular arrhythmias. *PLoS One* 2018;**13**:e0201910.

14. Todt H, Raberger G. Epicardial His bundle recordings in the guinea pig in vivo. *J Pharmacol Toxicol Methods* 1992;**27**:191-195.

15. Macías A G-GA, Moreno-Manuel AI, Cruz FM, Gutiérrez LK, García-Quintáns N, Roche-Molina M, Bermúdez-Jiménez FJ, Andrés V, Vera-Pedrosa ML, Martínez-Carrascoso I, Bernal JA and Jalife J. Kir2.1 dysfunction at the sarcolemma and the sarcoplasmic reticulum causes arrhythmias in a mouse model of Andersen–Tawil syndrome type 1. *Nature Cardiovascular Research* 2022;**1**:900–917.

16. Dhar Malhotra J, Chen C, Rivolta I, Abriel H, Malhotra R, Mattei LN, Brosius FC, Kass RS, Isom LL. Characterization of sodium channel alpha- and beta-subunits in rat and mouse cardiac myocytes. *Circulation* 2001;**103**:1303-1310.

17. Abriel H, Rougier JS, Jalife J. Ion channel macromolecular complexes in cardiomyocytes: roles in sudden cardiac death. *Circ Res* 2015;**116**:1971-1988.

18. Matamoros M, Perez-Hernandez M, Guerrero-Serna G, Amoros I, Barana A, Nunez M, Ponce-Balbuena D, Sacristan S, Gomez R, Tamargo J, Caballero R, Jalife J, Delpon E. Nav1.5 N-terminal domain binding to alpha1-syntrophin increases membrane density of human Kir2.1, Kir2.2 and Nav1.5 channels. *Cardiovasc Res* 2016;**110**:279-290.

19. Perez-Hernandez M, Matamoros M, Alfayate S, Nieto-Marin P, Utrilla RG, Tinaquero D, de Andres R, Crespo T, Ponce-Balbuena D, Willis BC, Jimenez-Vazquez EN, Guerrero-Serna G, da Rocha AM, Campbell K, Herron TJ, Diez-Guerra FJ, Tamargo J, Jalife J, Caballero R, Delpon E. Brugada syndrome trafficking-defective Nav1.5 channels can trap cardiac Kir2.1/2.2 channels. *JCI Insight* 2018;**3**.

20. Ponce-Balbuena D, Guerrero-Serna G, Valdivia CR, Caballero R, Diez-Guerra FJ, Jimenez-Vazquez EN, Ramirez RJ, Monteiro da Rocha A, Herron TJ, Campbell KF, Willis BC, Alvarado FJ, Zarzoso M, Kaur K, Perez-Hernandez M, Matamoros M, Valdivia HH, Delpon E, Jalife J. Cardiac Kir2.1 and NaV1.5 Channels Traffic Together to the Sarcolemma to Control Excitability. *Circ Res* 2018;**122**:1501-1516.

21. Park SS, Ponce-Balbuena D, Kuick R, Guerrero-Serna G, Yoon J, Mellacheruvu D, Conlon KP, Basrur V, Nesvizhskii AI, Jalife J, Rual JF. Kir2.1 Interactome Mapping Uncovers PKP4 as a Modulator of the Kir2.1-Regulated Inward Rectifier Potassium Currents. *Mol Cell Proteomics* 2020;**19**:1436-1449.

22. Moreno C, Prieto P, Macias A, Pimentel-Santillana M, de la Cruz A, Traves PG, Bosca L, Valenzuela C. Modulation of voltage-dependent and inward rectifier potassium channels by 15-epi-lipoxin-A4 in activated murine macrophages: implications in innate immunity. *J Immunol* 2013;**191**:6136-6146.

23. Steinegger M, Meier M, Mirdita M, Vohringer H, Haunsberger SJ, Soding J. HH-suite3 for fast remote homology detection and deep protein annotation. *BMC Bioinformatics* 2019;**20**:473.

24. Song Y, DiMaio F, Wang RY, Kim D, Miles C, Brunette T, Thompson J, Baker D. High-resolution comparative modeling with RosettaCM. *Structure* 2013;**21**:1735-1742.

25. Alford RF, Koehler Leman J, Weitzner BD, Duran AM, Tilley DC, Elazar A, Gray JJ. An Integrated Framework Advancing Membrane Protein Modeling and Design. *PLoS Comput Biol* 2015;**11**:e1004398.

26. Fleishman SJ, Leaver-Fay A, Corn JE, Strauch EM, Khare SD, Koga N, Ashworth J, Murphy P, Richter F, Lemmon G, Meiler J, Baker D. RosettaScripts: a scripting language interface to the Rosetta macromolecular modeling suite. *PLoS One* 2011;**6**:e20161.

27. Lomize MA, Pogozheva ID, Joo H, Mosberg HI, Lomize AL. OPM database and PPM web server: resources for positioning of proteins in membranes. *Nucleic Acids Res* 2012;**40**:D370-376.

28. Pravda L, Sehnal D, Tousek D, Navratilova V, Bazgier V, Berka K, Svobodova Varekova R, Koca J, Otyepka M. MOLEonline: a web-based tool for analyzing channels, tunnels and pores (2018 update). *Nucleic Acids Res* 2018;**46**:W368-W373.

29. Baker NA, Sept D, Joseph S, Holst MJ, McCammon JA. Electrostatics of nanosystems: application to microtubules and the ribosome. *Proc Natl Acad Sci U S A* 2001;**98**:10037-10041.

30. Dolinsky TJ, Nielsen JE, McCammon JA, Baker NA. PDB2PQR: an automated pipeline for the setup of Poisson-Boltzmann electrostatics calculations. *Nucleic Acids Res* 2004;**32**:W665-667.

31. Capra JA, Laskowski RA, Thornton JM, Singh M, Funkhouser TA. Predicting protein ligand binding sites by combining evolutionary sequence conservation and 3D structure. *PLoS Comput Biol* 2009;**5**:e1000585.
